# Mouse neuronal dendritic complexity and resilience to stress-induced depression in *Drosophila melanogaster* are enhanced by *Withania somnifera* alkaloids

**DOI:** 10.1016/j.jep.2025.120905

**Published:** 2025-11-21

**Authors:** Rudranil Dutta, Helen Holvoet, Luke Marney, Kadine Cabey, Cody Neff, Mikah Brandes, Jonathan Zweig, Jesus Martinez, Jaewoo Choi, Christine McClure, Md Nure Alam, Liping Yang, Burkhard Poeck, Jan F. Stevens, Doris Kretzschmar, Nora E. Gray, Roland Strauss, Claudia S. Maier, Amala Soumyanath

**Affiliations:** a BENFRA Botanical Dietary Supplements Research Center, Oregon Health & Science University, Portland, OR, 97239, USA; b Department of Chemistry, Oregon State University, Corvallis, OR, 97331, USA; c Institute for Developmental Biology and Neurobiology, Johannes Gutenberg-Universität Mainz, Hanns-Dieter-Hüsch-Weg 15, 55128, Mainz, Germany; d Department of Neurology, Oregon Health & Science University, Portland, OR, 97239, USA; e Linus Pauling Institute, Oregon State University, Corvallis, OR, 97331, USA; f Department of Pharmaceutical Sciences, Oregon State University, Corvallis, OR, 97331, USA; g Oregon Institute of Occupational Health Sciences, Oregon Health & Science University, Portland, OR, 97239, USA

**Keywords:** Ashwagandha, *Drosophila*, Primary neurons, Depression, Withanolides, Alkaloids, Acetyltropine

## Abstract

**Ethnopharmacological relevance:**

*Withania somnifera* (WS), also known as ashwagandha, is used in traditional Ayurvedic medicine for its rejuvenating properties. While most research has focused on withanolides as primary bioactive constituents, this study highlights the potential role of alkaloids in the biological effects of WS.

**Aim of the study:**

To explore compounds responsible for bioactivity of WS on dendritic complexity in mouse primary neurons and on stress-related behaviors in *Drosophila melanogaster*.

**Materials and methods:**

An aqueous extract was prepared from dried root of WS grown in Central Oregon, and fractionated into ethanol insoluble, polar, and non-polar fractions. The preparations were tested for effects on dendritic complexity of mouse primary hippocampal neurons, and on stress-induced behavioral changes in *Drosophila melanogaster* flies. The composition of WS extract and fractions was examined using liquid chromatography coupled to high-resolution or multiple reaction monitoring mass spectrometry (LC-HRMS/MS or LC-MRM-MS).

**Results:**

WS root aqueous extract and its polar fraction enhanced dendritic complexity in mouse primary hippocampal neurons and significantly improved stress-related behavior in *Drosophila*, whereas the withanolide-rich, non-polar fraction did not. The bioactive, polar fraction contained alkaloids including acetyltropine, which was then confirmed as a major, bioactive alkaloid in WS root and differentiated from its isomer acetyl exotropine by its LC-MRM-MS retention time and 2D-NMR.

**Conclusions:**

These findings highlight a role for alkaloids, including acetyltropine, as potential active constituents of WS associated with neuroprotective and anti-stress effects traditionally attributed to this plant. Results support continued evaluation of additional WS active compounds beyond withanolides.

## 1. Introduction

*Withania somnifera* (L.) Dunal (family Solanaceae), commonly known as ashwagandha (WS), is a botanical with a long history of medicinal use as a “rasayana” or rejuvenating herb in the Ayurvedic system of India (Gautam and Pandey, 2020; Singh et al., 2011). The roots and leaves are the most common parts of the plant used traditionally (Nadkarni, 1976; Kapoor, 1990) although medicinal uses of the flowers and seeds have also been reported (Singh et al., 2011). Uses of WS in Ayurvedic medicine include the relief of insomnia, senile and general debility, nervous exhaustion, brain fag, loss of memory and loss of muscle energy (Nadkarni, 1976; Kapoor, 1990). In recent years, the use of commercial WS products as botanical dietary supplements has increased considerably e.g. in the United States (Smith et al., 2021; Smith et al., 2024). This is due primarily to the reputation of WS as an adaptogen (Gautam and Pandey, 2020) i.e. a substance that improves the body’s ability to adapt to stress (Panossian et al., 2021). The widespread and growing use of WS products to aid sleep and stress management (Sprengel et al., 2025) highlights the need for ongoing research evaluating the benefits and risks of this botanical.

Research studies of WS are abundant in the literature. Pre-clinical studies have identified anti-stress, anti-anxiety, sleep-promoting, and neuroprotective effects of WS preparations in various animal models (Speers et al., 2021; Zahiruddin et al., 2020). Thirty or more clinical trials of ashwagandha have been performed examining different biological end points (Tandon and Yadav, 2020). Systematic reviews and meta-analysis of these clinical trials, while limited by small sample sizes and heterogeneity between studies, have found evidence that WS can reduce anxiety and stress (Akhgarjand et al., 2022), improve sleep (Cheah et al., 2021), and enhance various aspects of cognitive function (Ng et al., 2020).

While WS has been widely studied, one challenge in utilizing these studies to inform evidence based phytotherapy with WS is that the preparations investigated vary widely between studies and are frequently inadequately described or characterized (Speers et al., 2021). Rigorous preclinical studies and clinical trials on well characterized products are therefore still needed for WS. Optimization of product composition for particular therapeutic effects, based on a knowledge of the relevant active compounds, is a key part of performing rigorous clinical evaluations of natural products (Sorkin et al., 2020; Weber and Hopp, 2020).

Studies on bioactive compounds of WS have focused on withanolides, a group of naturally occurring steroidal lactones, and withanosides, which are their glycosidic forms (Durg et al., 2015). Withanolides are abundant in WS, and over 40 have been isolated from the roots, leaves and fruit of WS (Lavie et al., 1965; Gómez Afonso et al., 2023). Withaferin A is the most studied withanolide, with reported anticancer effects in pre-clinical models, as well as demonstrated anti-inflammatory, anti-diabetic, neuroprotective, cardioprotective, and bone-building effects (Kumar et al., 2023). There is also pre-clinical evidence for neuroprotective, anti-inflammatory, anti-oxidant and anti-depressive effects of other withanolides, including withanolide A (Tiwari et al., 2023; Tiwari et al., 2022; Akhoon et al., 2016; Baitharu et al., 2014; Naβ et al., 2021), withanone (Dar et al., 2017; Konar et al., 2011; Pandey et al., 2018; Naβ and Efferth, 2021), sominone (Joyashiki et al., 2011; Tohda and Joyashiki, 2009; Kuboyama et al., 2006), and 12-deoxywithastramonolide (Chandan et al., 2021; Yang et al., 2014). However, in addition to withanolides, a variety of other phytochemicals have been isolated from WS, including flavonoids, steroids, alkyl ferulates, alkaloids and other nitrogen-containing compounds (Bashir et al., 2023; Sonar et al., 2019; Tetali et al., 2021). Several lines of pre-clinical evidence suggest that non-withanolide compounds also contribute to the effects of WS (Kaushik et al., 2017; Maccioni et al., 2022; Dey et al. 2018; Sonar et al., 2019; Singh et al., 2011; Holvoet et al., 2022).

An earlier study by our group compared the effects of two WS extracts differing in withanolide content on stress induced depression-like behavior in *Drosophila melanogaster* (Holvoet et al., 2022). This *Drosophila* stress model (Ries et al., 2017) can be used to assess the behavioral impact of chronic stress and the potential functional protective effects of test compounds to modulate stress-related behaviors. This model is also used to study conserved neurobehavioral pathways relevant to mood and resilience. Our results demonstrated that WS water extract could provide resilience against stress-induced depression-like behavior in *Drosophila*, and that WS compounds other than withanolides appeared to be responsible for these effects (Holvoet et al., 2022). In the present study, we fractionated the active WS extract into subgroups of components in order to further explore the nature of the bioactive compounds present.

To expand our understanding of WS’s neuroactive properties, we added an assay involving mouse primary hippocampal neuron cultures. The measurement of dendritic complexity of murine primary neurons in cell culture assay offers an *in vitro* model of mammalian neuronal growth (Chen et al., 2013) that relates to the capacity to form synapses. These cultures can be used to directly measure the impact of natural products on neuronal morphology and development (Moise et al., 2024). Reduced hippocampal dendritic arborization (complexity) can result from chronic stress and accompanies depressive-like symptoms in mice (Hu et al., 2022; Gilabert-Juan et al., 2017). Therefore, this model can be used to explore the effects of WS on a property of mammalian neurons relevant to its effects on stress-induced depressive-like behavior in *Drosophila*.

In order to investigate the nature of WS constituents associated with the observed biological activities, we combined the *Drosophila* behavioral tests and mouse primary neuronal bioassay with chemical profiling of the fractions. Liquid chromatography in conjunction with high resolution tandem mass spectrometry (LC-HRMS/MS) has proven a valuable tool for the untargeted phytochemical analysis of botanical extracts including ashwagandha (Marney et al., 2024; Ha et al., 2022; Abraham and Kellogg, 2021). Chemical profiling by mass spectrometry (metabolomics) provides comprehensive information on metabolites present in complex botanical extracts (Kellogg et al., 2016). This approach also aids in identifying previously uncharacterized compounds that may contribute to the plant’s therapeutic effects. By integrating MS-based phytochemical profiling with bioassay-guided fractionation, bioactive leads can be more quickly and precisely linked to specific molecular signatures (Alcázar Magaña et al., 2024; Kellogg et al., 2016).

In addition, accurate quantification of selected phyto-constituents, once identified, can be achieved by tandem mass spectrometers that perform analyses in multiple reaction mode (MRM) mode, in which a precursor ion and its product ions are specified and monitored. LC-MRM-MS enables more selective quantitative analysis compared to single product ion monitoring and chemical profiling studies. This method has previously been applied to the measurement of withanolides in plant extracts and mammalian biological samples (Speers et al., 2024; Marney et al., 2024).

In the present study, we used LC-HRMS/MS to explore the chemical composition of the WS extracts and fractions that we tested for biological activity in *Drosophila* flies and in mouse primary hippocampal neurons. This allowed us to define the major phytochemical groups in active and inactive extracts. We also developed and validated an LC-MRM-MS method for the accurate quantitation of selected alkaloids that were found in the active fractions.

## 2. Materials

### 2.1. WS raw materials, extracts, fractions and compounds

Ashwagandha root grown at Oregon’s Wild Harvest (OWH; Redmond, OR, USA) was obtained in dried, powdered form (batch # 201000162) from the company. The root material was authenticated as *Withania somnifera* by comparing its thin-layer chromatographic profile (Raichura et al., 2025) with that of authentic *Withania somnifera* root reference material from the American Herbal Pharmacopoeia (AHP Batch # 4746) and by genetic testing as previously described (Marney et al., 2024). Voucher samples of the original root are stored at the BENFRA Laboratories at Oregon Health & Science University (OHSU; code name BEN-WS-8) and at the Herbarium at Oregon State University (OSU; Code OSC-V-265405). Solvents, acids and alkalis used for preparing ashwagandha extracts and fractions were purchased from Fisher Scientific (Hampton, NH, USA). Withanolides (>95 % purity) were purchased from the following sources: Withanone, withanoside IV, withanoside V from TransMIT (Gieβen, Germany), withaferin A from ChemFaces (Wuhan, China), withanolide A from Chromadex (now Niagen; Tustin, CA, USA) and withanolide B and 12-deoxywithastramonolide from Cayman Chemicals (Ann Arbor, MI, USA). Tropine was purchased from Cayman Chemicals. Identity and purity of the compounds were verified by liquid chromatography coupled to high resolution tandem mass spectrometry (LC-HRMS/MS) and 1H nuclear magnetic resonance (NMR) analysis (data not shown).

### 2.2. Mouse hippocampal primary neuron bioassay

Primary neurons were isolated (section 3.2.1) from embryonic offspring of C57BL6 mice purchased from Jackson Laboratories (Bar Harbor, ME, USA). MEM medium, Anti-anti, GlutaMAX and neurobasal medium were purchased from GIBCO/Life Technologies (Waltham, MA, USA). Fetal bovine serum (FBS) was purchased from Atlanta Biologicals (Flowery Branch, GA, USA). Glucose, cytosine β-D-arabinofuranoside hydrochloride (AraC) and AntiMAP2B antibody were purchased from Sigma-Aldrich (St. Louis, MO, USA). GS21 neural supplement was purchased from ThermoFisher (Waltham, MA, USA) and goat anti-mouse IgG1-Cy3 was purchased from Jackson ImmunoResearch (West Grove, PA, USA).

### 2.3. Chemicals and solvents used for LC-MS

LC-MS grade water, acetonitrile and formic acid were purchased from Fisher Scientific (Hampton, NH, USA). Deuterated authentic standard of atropine-d_5_ was purchased from Cayman Chemical (Ann Arbor, MI, USA) and was used as a heavy labeled internal standard (purity ≥95 %).

### 2.4. Acetoxytropane isomers

Acetyltropine and acetyl exotropine were synthesized by the Medicinal Chemistry Core at Oregon Health & Science University (Supplemental material, Section S1).

## 3. Methods

### 3.1. Ashwagandha root extracts and fractions

#### 3.1.1. Ashwagandha root hydroethanolic extract

A 70 % ethanol extract (WSE-2) of ground, dried ashwagandha root material (BEN-WS-8) was prepared as previously described (Holvoet et al., 2022). Briefly, the plant material was boiled under reflux with 70 % ethanol (12.5 mL solvent/g plant material) for 90 min. Following filtration to remove plant debris, ethanol was removed from the filtered extract under vacuum, and the remaining aqueous solution frozen and lyophilized to yield a dry powder (WSE-2, 5.4 % of starting root material). Liquid chromatography-high resolution mass spectrometry (LC-HRMS/MS) fingerprints were acquired for digital archiving of extracts to accompany voucher samples. A 2D ion map of WSE-2 (which was used in this study) is provided in supplemental material (Fig. S1B).

#### 3.1.2. Ashwagandha root water extract

Three batches of hot water extract (WSAq-9, WSAq-16 and WSAq-17) were prepared from ashwagandha root (BEN-WS-8) using a similar method for all 3 batches (Holvoet et al., 2022). Ground root material was boiled under reflux with water (12.5 mL water/g plant material) for 90 min. While still warm, the plant extract was decanted through a kitchen sieve to remove larger plant debris. The liquid was transferred to multiple plastic tubes (Falcon, 50 mL tubes) and centrifuged (Beckman GS-6R; 3750 rpm) to precipitate fine particles, and the supernatant filtered through filter paper (Whatman; Grade 1) under vacuum. The filtrate was lyophilized to give a light brown, dry powder (yields WSAq-9, 8.4 %; WSAq-16, 7.5 %; WSAq-17, 9.4 % w/w of starting root material). LC-HRMS/MS fingerprints were acquired for digital archiving of extracts to accompany voucher samples. A 2D ion map of WSAq-9 generated using LC-HRMS/MS is provided in supplemental material (Fig. S1A), 2D ion maps of WSAq-16 and WSAq17 (Fig. 4) are described under section 4.5.

#### 3.1.3. Fractionation of ashwagandha root water extracts

##### First fractionation (WSAq-16; Fig. S2):

Dried WSAq-16 (6.08 g) was distributed between 4 centrifuge tubes (approximately 1.5 g per tube). Ethanol (25 mL) was added to each tube; the mixture shaken well, centrifuged (Beckman GS-6R; 3750 rpm) and the supernatants collected and combined. This was repeated two more times with fresh ethanol to give a total extraction volume 300 mL for the 6.08 g of WSAq-16. The sediments from the centrifuge tubes were combined and air dried on a Petri dish in the fume hood to yield the ethanol-insoluble fraction (WSF-9; 5.81g). The ethanolic supernatants were filtered (Whatman Grade 1 paper) to remove fine particles, and the filtrate was dried on a rotary evaporator under vacuum at 60 °C. The residue in the round-bottomed flask was redissolved in water (60 mL) and transferred to a separating funnel. Once cool, the water layer was extracted with methylene chloride (3 × 60 mL). The combined methylene chloride layers were designated WSF-10, and the water layer was designated WSF-11. Residual water in the WSF-10 methylene chloride layer was removed by addition of anhydrous magnesium sulfate. The liquid was then filtered (Whatman Grade 1 paper), evaporated to dryness under vacuum, reconstituted in water (15 mL) and dried by lyophilization to yield the ethanol-soluble, non-polar methylene chloride fraction (WSF-10; 7.0 mg). The extracted water layer from the separating funnel (WSF-11) was processed in a rotary evaporator under vacuum at 40 °C to remove residual methylene chloride after which it was lyophilized to yield the ethanol-soluble, polar, water fraction (WSF-11; 41.7 mg).

##### Second fractionation (WSAq-17; Fig. S3A):

A similar process was followed using approximately double the quantities as for the first fractionation. Dried WSAq-17 (12.0 g) was shaken with ethanol (600 mL) and yielded an ethanol-insoluble fraction (WSF-12; 11.63 g). The dried, ethanol-soluble fraction was partitioned between water (120 mL) and methylene chloride (3 × 120 mL) to yield an ethanol-soluble, non-polar, methylene chloride fraction (WSF-13; 29 mg) and an ethanol-soluble, polar, water fraction (WSF-14; 171.4 mg).

#### 3.1.4. Ashwagandha root alkaloidal extract (Figure S4)

Ashwagandha root powder (BEN-WS-8; 90 g) was stirred with dilute sulfuric acid (0.05M; 900 mL) at 50 ± 5 °C for 30 min. The mixture was cooled on ice and the plant material was allowed to settle. The acidic liquid extract was decanted, and centrifuged (Beckman GS-6R; 3750 rpm) to sediment remaining plant material. The supernatants were combined and filtered (Whatman Grade 1 paper) to yield an aqueous acidic extract (WSAq-15; approximately 500 mL). An aliquot of this extract (10 mL) was neutralized with ammonium hydroxide (13.5 M) and lyophilized (WSAq-15; 362.4 mg). This was used to estimate the amount of extracted material in the bulk of the WSAq-15 solution (18.12 g).

The main acidic extract (WSAq-15; 500 mL) was transferred to a separating funnel and extracted with methylene chloride (3 × 250 mL). Alkaloids, if present, would be expected to remain in the acidic water layer as polar salts, whereas less polar compounds would partition favorably into the organic methylene chloride layer. The organic layers were combined and residual water removed by addition of anhydrous magnesium sulfate. The dried methylene chloride fraction was then filtered (Whatman Grade 1 paper), evaporated to dryness under vacuum, redissolved in water (20 mL) and dried by lyophilization to yield an organic fraction (WSF-4; 59.2 mg).

The pH of the aqueous layer from this first solvent:solvent partition step was then raised from acidic (pH < 3) to alkaline (pH > 10) by addition of ammonium hydroxide (13.5 M) in order to convert any alkaloidal salts present to their less polar free base forms. A second extraction step with methylene chloride (3 × 250 mL) was carried out to extract these alkaloidal bases. Residual water in the combined methylene chloride layers was again removed by additional anhydrous magnesium sulfate. This second, dried methylene chloride fraction was filtered (Whatman Grade 1 paper), evaporated to dryness under vacuum and reconstituted in sulfuric acid (0.05M; 20 mL) to facilitate removal from the flask. This solution was neutralized to pH 7 with ammonium hydroxide (13.5 M) and dried by lyophilization to yield an alkaloidal fraction (WSF-5; 354.1 mg). The twice extracted water layer was neutralized to pH 7 with concentrated sulfuric acid (18.4 M) and lyophilized to yield the dried residual water fraction (WSF-6; 7.30g).

#### 3.1.5. Voucher samples

Voucher samples of all the extracts and fractions are stored at the corresponding author’s laboratory at OHSU under the code names listed above, with the prefix “BEN” to indicate their origin at the BENFRA Center.

### 3.2. Assessing effects on dendritic complexity in mouse hippocampal neurons

#### 3.2.1. Primary hippocampal neuron isolation and culture

Mice were housed in an AALAC certified facility and maintained in a climate-controlled environment with ad libitum diet and water on a 12-h light/12-h dark cycle and fed a Pico Lab Rodent Diet 5LOD. All procedures were conducted in accordance with the NIH Guidelines for the Care and Use of Laboratory Animals and were approved by the institutional Animal Care and Use Committee of OHSU (Protocol #4469–23).

Hippocampal neurons were isolated from C56BL6 embryonic mice, based on the methods previously described (Kaech and Banker, 2006). Embryos were harvested at 18 days of gestation from anesthetized females and hippocampi dissected, gently minced, trypsinized, and triturated to generate suspensions of dispersed neurons. Neurons were plated at a density of 130,000 cells in 60 mm dishes each containing 3 poly-l-lysine-coated nitric acid treated glass coverslips with paraffin wax spacers in MEM medium containing 5 % FBS, 1 × Anti-Anti and 0.6 % glucose. After 3 h, the coverslips were flipped into 60 mm dishes containing mouse neural stem cell-derived glial cells (provided by Dr. Gary Banker, Jungers Center, OHSU) and maintained in 6 mL Neurobasal Medium supplemented with 1 × GlutaMAX, 1 × Anti-Anti and 1 × GS21 neural supplement. Dishes were fed every week by removing 1 mL of the culture medium and adding 1 mL fresh Neurobasal medium that included GlutaMAX, Anti-Anti and Neuronal Culture Medium Supplement, with the first feed at 5 days *in vitro* (DIV) containing 6 μM (1 μM final) cytosine β-D-arabinofuranoside hydrochloride.

After 14 days *in vitro* (DIV) cells were treated with test materials. In an initial experiment, cells were treated with WS aqueous and hydroethanolic extracts WSAq-9 (50 and 100 μg/mL) or WSE-2(50 and 100 μg/mL), or a mixture of withanolides with concentrations corresponding to their content in WSAq-9, 100 μg/mL (withanolide A, 100 ng/mL; withanolide B, 0.3 ng/mL; withaferin A, 20 ng/mL; withanone, 110 ng/mL; withanoside IV,20 ng/mL; withanoside V, 6.9 ng/mL; 2-deoxywithastramonolide, 3.9 ng/mL). A follow-up experiment tested a similar aqueous extract WSAq-16 (100 μg/mL) and its fractions at concentrations equivalent to WSAq-16, μg/mL (WSF-9, 95.5 μg/mL; WSF-10, 0.12 μg/mL; WSF-11; 0.69 μg/mL) as shown in Table S1. Changes in complexity were assessed by comparing the treatment groups to control cells that were treated with an equivalent volume of water.

#### 3.2.2. Assessment of dendritic complexity

At 19 DIV coverslips were fixed in 4 % and stained with Anti-MAP2B and Goat anti-mouse IgG1-Cy3. Immunostained neurons were imaged with a Zeiss ApoTome2 microscope and blinded Sholl analyses were performed to assess dendritic complexity using the Fiji platform. Analysis consisted of quantifying the number of intersections of dendritic processes with concentric circles drawn at 10 μm intervals from the cell body out to 300 μm. Plotting the number those intersections as a function of distance from the cell body generates a curve and results are represented as area under the curve. Thirty isolated, non-overlapping cells were analyzed per coverslip. Complexity data was pooled across 3 independent experiments (3–5 coverslips per treatment condition in each experiment) providing at least 300 cells per treatment condition.

#### 3.2.3. Statistical analysis

Statistical analyses were done using GraphPad Prism. ANOVAs were used to compare multiple groups with Tukey’s post-hoc pairwise testing. Significance levels are indicated by asterisk with *p < 0.05, **p < 0.01, ***p < 0.001. Detailed raw data summaries and statistical comparisons are available in Supplementary Table S2.

### 3.3. Assessing effects on stress-induced behavior in Drosophila melanogaster

#### 3.3.1. Drosophila strain and food preparation

*Drosophila melanogaster* wild-type Canton-Special strain (CS, FBst0064349) was provided by M. Heisenberg and used for all experiments. Flies were kept on standard *Drosophila* food (cornmeal/soy/yeast/molasse) at 25 °C under a 14:10h light:dark cycle. Supplemented food wa prepared by mixing the WS extracts or fractions, provided as 10x aqueous stock solutions, or by dissolving solid compounds in 70 % EtOH or ddH_2_O to create stock solutions, which were then mixed into liquid standard *Drosophila* food. To obtain untreated controls, the same amount of water, or 70 % ethanol solution, as used for the test additives was added to the food. We previously showed that feeding the flies WS extracts did not affect their consumption (Holvoet et al., 2022).

#### 3.3.2. WS test materials

*Drosophila* flies were treated with various test substances derived from WS (concentrations given as amount/g of food). A mixture of withanolides was tested corresponding to their concentration in WSE-2 (2 mg/g): withanolide A, 16.64 μg/g; withanolide B, 0.06 μg/g; withaferin A, 3.74 μg/g; withanone, 18.46 μg/g; withanoside IV 3.98 μg/g; withanoside V, 1.49 μg/g; 2-deoxywithastramonolide, 0.59 μg/g. The mixture was tested with or without addition of the alkaloid tropine (6.85 μg/g). Flies were also treated with WS extracts and fractions: WSAq-17 (0.5 mg/g), WSF-12 (0.5 mg/g), WSF-13 (1 μg/g), WSF-14 (7 μg/g), WSF-5 (10 μg/g), and with acetyltropine (0.4 μg/g). Test concentrations of the fractions corresponded to their weights relative to the parent extract as shown in Table S1.

#### 3.3.3. Drosophila stress protocol

Flies were collected after eclosion and their wings shortened when they were 2–3 days old to prevent flying. Groups of 10–20 flies were then placed on standard food or food supplemented with WS test materials for 10 days. Vials were replaced with fresh ones on day 5 and day 10. When 13d old, stress was induced with repetitive phases of 300Hz vibrations using cohorts of 10–20 flies confined to empty, narrow tubes during daytime as described previously (Ries et al., 2017) After the stress application, flies were transferred to standard food or food supplemented with test compound for the night. For the non-vibrated control (non-vib.), flies were confined to the same empty, narrow tubes and placed next to the vibrating device for the same amount of time. For determining the prophylactic effects of the WS extracts (pro.), flies previously receiving supplemented food were kept on standard food during the three days of stress application. For continuous treatment (con.), flies were returned to supplemented food in each rest period of the stress protocol.

#### 3.3.4. Drosophila gap climbing assays

To determine the motivation of flies to initiate climbing attempts at a 4.5-mm-wide gap before the stress is applied, the flies were tested in a pre-test after the 10d of food administration with and without the WS extract or compounds. Each fly was allowed to perform ten approaches and only flies that showed four or more climbing attempts in this pre-test (PT) were included in the stress protocol. After the stress application, the flies had a short resting period on standard food before performing the post-stress tests (T1) on day 13. An attempt to climb the gap was defined by the stereotypical leg-over-head behavior as described previously (Ries et al., 2017).

#### 3.3.5. Drosophila stop-for-sweet (S4S) assay

Flies were subjected to the same feeding and stress protocol as described above. However, after the last stress application the flies were placed in empty vials to deprive them of food for at least 8 h. The S4S paradigm was then performed similar to the protocol in. Individual flies were confined to rectangular chambers of 55 × 20 mm^2^ that were cut out of a 3-mm-thick white foam board, with a bottom of clear plastic. The chambers were then covered with a cut-to-size filter paper on which a 5-mm-wide trace of glycerol (99.5 %) had been applied with a fine paintbrush along the midline of the paper. The flies were shaken to the bottom of the chamber and the chamber turned (110°–120°) to make the flies walk at a 90° angle upwards on the filter paper. Each fly was observed whether it stopped and extended its proboscis at the glycerol or kept walking up the filter paper. After proboscis extension or continuous walking was observed, the fly was immediately shaken to the bottom again to prevent ingestion, and the test repeated ten times for each fly.

#### 3.3.6. Statistical analysis

For both the gap-climbing and S4S assays, comparative statistical analyses were carried out using RStudio (version 2022.10.0 + 353). Shapiro-Wilk’s tests were used to test for normal distribution and a pairwise *t*-test with Bonferroni-Holm correction was used for multiple comparisons where appropriate. For nonparametric data, pairwise Wilcoxon-test was used with built-in Bonferroni-Holm correction to compare three or more experimental groups/conditions against each other. Detailed raw data summaries and statistical comparisons are available in Supplementary Table S3A–S3E.

### 3.4. Chemical profiling of WS extracts and fractions using liquid chromatography coupled to high resolution tandem mass spectrometry (LC-HRMS/MS)

#### 3.4.1. LC-HRMS/MS analysis

Chromatography was performed on a Phenyl-3 column (GL Sciences 2.1 × 100 mm, 1.8 μm) with gradient elution. Mobile phases consisted of solvent A (0.1 % formic acid in water, v/v) and solvent B (0.1 % formic acid in acetonitrile, v/v). The LC gradient was 0.00–0.10 min: 30 % B; 0.10–8.00 min: 50 % B; 8.00–8.50 min: 98 % B; 8.50–10.00 min: 98 % B; 10.00–10.50 min: 30 % B; 10.50–12.00 min: 30 % B. The flow rate was 0.6 mL/min, column oven was maintained at 60 °C, and injection volume was 3 μL.

Mass spectrometric data were acquired using a SCIEX 7600 ZenoTOF mass spectrometer with TurboIonSpray ionization in positive mode. MS1 (TOF-MS) data were collected over the m/z range 50–1200. All data was acquired using information-dependent acquisition mode with a maximum of 30 precursor candidates per cycle. The following settings were used: MS1 range: 50–1200 Da, and collision energy 12 eV. The MS/MS scans (TOF-MS/MS) used a fixed collision energy of 35 V, 0.02 s accumulation time, and Zeno pulsing enabled with a 20,000 cps threshold. This system utilizes automated mass calibration to ensure accuracy and performance throughout data acquisition and allows acquiring data with a resolving power of ~42,000 full-width at half-mast (FWHM).

#### 3.4.2. Sample preparation

Extracts and fractions were generated from WS root as described earlier (Section 3.1; Figs. S2–S4). An appropriate amount of LC-MS grade methanol:water (70:30) with 0.1 % formic acid (FA) was added to each of the WS materials to make a 10 mg/mL solution in eppendorf tubes. For BEN-WS-8 root powder, 10 mg of solid was measured and 1 mL of LC-MS grade water +0.1 % FA was added to make a 10 mg/mL solution in eppendorf tube. Extracts, fractions and BEN-WS-8 10 mg/mL mixtures were vortexed for 30 s, and sonicated for 15 min at room temperature. The vortexing and sonication steps were repeated for a second time. The mixtures were centrifuged (10 min at 14000 g), the supernatant was collected, and diluted to the equivalent of 0.25 mg/mL starting material by adding the appropriate volume of LC-MS grade water + 0.1 % FA. Prepared samples were stored at – 80 °C if not analyzed immediately.

#### 3.4.3. Data processing

LC-HRMS/MS 2D ion maps were prepared in R (http://www.r-project.org) (Team, R. Core, 2021) using an in-house script with ggplot2 plotting tools (code available upon request) (Wickham, 2009)

### 3.5. Analysis of acetyltropine and related alkaloids using liquid chromatography coupled to multiple reaction monitoring mass spectrometry (LC-MRM-MS)

#### 3.5.1. LC-MRM-MS analysis

A Waters Xevo TQ-XS mass spectrometer coupled to a Waters Acquity UPLC I-Class system (Waters, Milford, MA) was used for LC-MRM-MS of acetyltropine in BEN-WS-8 and its extracts and fractions. Chromatography was performed on an Agilent Zorbax eclipse plus C18 column (50 × 4.6 mm, 1.8 μm particle size) with a 17-min gradient containing 0.1 % FA in water (mobile phase A) and 0.1 % FA in acetonitrile (mobile phase B). The gradient was as follows: 0–3 min, 5 % B; 3.01–5.00 min, 5–10 % B; 5.01–8.00 min, 10–50 % B; 8.01–9.00 min, 50–98 % B; 9.01–14.99 min, 98 % B; 15.00–17.00 min, 5 % B. The column was held at 55 °C, the flow rate was 0.6 mL/min. Injection volume was 1 μL for each sample. All experiments were performed in positive electrospray ionization (ESI+) mode. Desolvation gas flow and cone gas flow were 1000 L/h and 150 L/h, respectively. Spray voltage and desolvation gas temperature were kept constant and set at 2300 V and 600 °C. Tandem mass spectrometric transitions were used for multiple reaction monitoring (MRM) of acetyltropine, internal standard (atropine-d_5_), and related alkaloids. Table S4 summarizes optimized MRM transitions, cone voltages and collision energies used to analyze the alkaloids of interest i.e. acetyltropine, atropine-d5, pseudotropine, tigloyloxytropane and tropine (Fig. S6). The development and validation of this method is described in supplementary section S2.2.

#### 3.5.2. Sample preparation

Dried BEN-WS-8 powder (~10 mg) was suspended in 1 mL water containing 0.1 % FA and atropine-d_5_ (0.001 μg/mL) to make a 10 mg/mL solution. The WS extracts and fractions had were suspended in appropriate volume of the same solution to produce a 10 mg/mL solution. After vortexing (30 s), samples were sonicated at room temperature (15 min). The vortexing and sonication steps were repeated after which samples were centrifuged (14,000 g, 10 min), and the supernatant transferred to a new Eppendorf tube. All samples were diluted appropriately with water containing 0.1 % formic acid and the internal standard atropine-d_5_ (0.001 μg/mL). Dried WS powder as well as fractions were diluted to 0.25 mg/mL before LC-MRM-MS analysis. Since different weights of dried fractions were obtained, the volume was varied for dilution.

#### 3.5.3. Data processing

TargetLynx Application Manager, an option within Waters MassLynx software, was applied to process all raw data files and provided accurate quantification results for the targeted compounds.

## 4. Results

### 4.1. Withanolide content in WS aqueous and hydroethanolic extracts

Initially, two types of parent extract, an aqueous extract (WSAq-9) and a hydroethanolic extract (WSE-2) were prepared from an authenticated sample of WS root (BEN-WS-8) grown in Redmond, Oregon, USA. The content of selected withanolides, for which commercial reference standards were available, in these two extracts was measured by LC-MRM-MS and has been previously reported (Holvoet et al., 2022). These results showed that the withanolides (withanolide A, withanolide B, 12-deoxywithastramonolide, withaferin A, withanone, withanoside IV, withanoside V) were enriched 3–10 fold in the hydroethanolic extracts (WSE-2) compared to the water extract (WSAq-9).

### 4.2. WS water extract and a polar fraction increase dendritic complexity, whereas a hydroalcoholic extract and a withanolide mixture do not

**Fig. 1.**
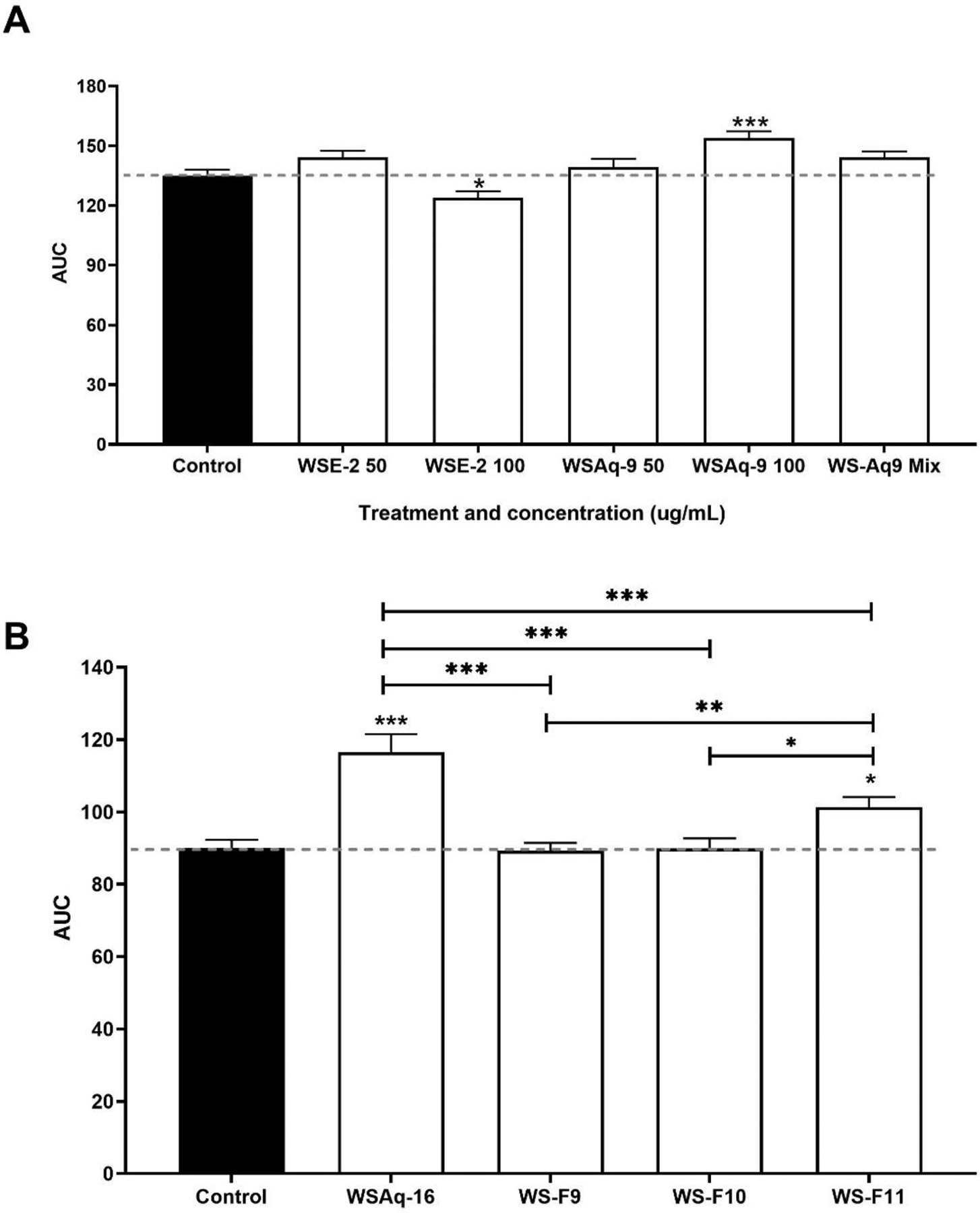
WS aqueous extracts and a polar fraction of the aqueous extract increased dendritic arborization in mouse hippocampal primary neurons. Area under the curve (AUC) generated by Sholl analysis of neurons (section 3.3.2) is a measure of dendritic complexity (A) Comparison of dendritic complexity of mouse primary hippocampal neurons after treatment with aqueous (WSAq-9; 50 and 100 μg/mL) or hydro-ethanolic (WSE-2; 50 and 100 μg/mL) extracts of WS root, or a mixture of withanolides (WSAq-9 mix) at the concentration present in WSAq-9, 100 μg/mL. (B) Comparison of dendritic complexity after treatment with an aqueous extract (WSAq-16; 100 μg/mL) of WS root, as well as equivalent concentrations of an ethanol insoluble fraction (WSF-9; 95.5 μg/mL), a non-polar fraction (WSF-10; 0.12 μg/mL) and a polar fraction (WSF-11; 0.69 μg/mL) derived from WSAq-16. Treatment with WS root aqueous extracts WSAq-9 and WSAq-16 (100 μg/mL) significantly increased the area under AUC of the hippocampal mouse neurons. WSF11 (0.69 μg/mL) also increased complexity but to a lesser extent than WSAq-16. Neither WSF-9 nor WSF-10 influenced complexity in primary mouse hippocampal neurons. Error bars reflect standard error of the mean (S.E.M) *p < 0.05; **p < 0.01; ***p < 0.001 compared to control unless otherwise indicated.

Dendritic complexity of cultured primary neurons was assessed by Sholl analysis (section 3.2.2). There was no observable toxicity in the neurons with any of the treatment conditions. In an initial experiment (Fig. 1A), treatment with WS root aqueous extract (WSAq-9; 100 μg/mL) significantly increased dendritic complexity, whereas WS root ethanolic extract (WSE-2; 50 or 100 μg/mL) did not. Similarly, a mixture of seven commercial withanolides mimicking their concentrations in WSAq-9 did not increase dendritic complexity. This result, along with the observation that WSE-2 has a higher content of withanolides than WSAq-9 (Holvoet et al., 2022) suggested that withanolides may not be the active compounds in WSAq-9.

To explore the nature of the active compounds, a new batch of WS aqueous extract, WSAq-16 was fractionated and the fractions (WSF 9,10 and 11) explored for the ability to increase dendritic arborization (Fig. 1B). WSAq-16 (100 μg/mL) significantly increased dendritic complexity of the hippocampal mouse neurons as previously observed with WSAq-9. Only one fraction of WSAq-16, WSF-11 also increased dendritic complexity but to a lesser extent than WSAq-16. Neither WSF-9 nor WSF-10 influenced complexity in primary mouse hippocampal neurons.

### 4.3. Protective effects of WS on stress-induced behavioral changes in Drosophila melanogaster are not associated with withanolides

**Fig. 2.**
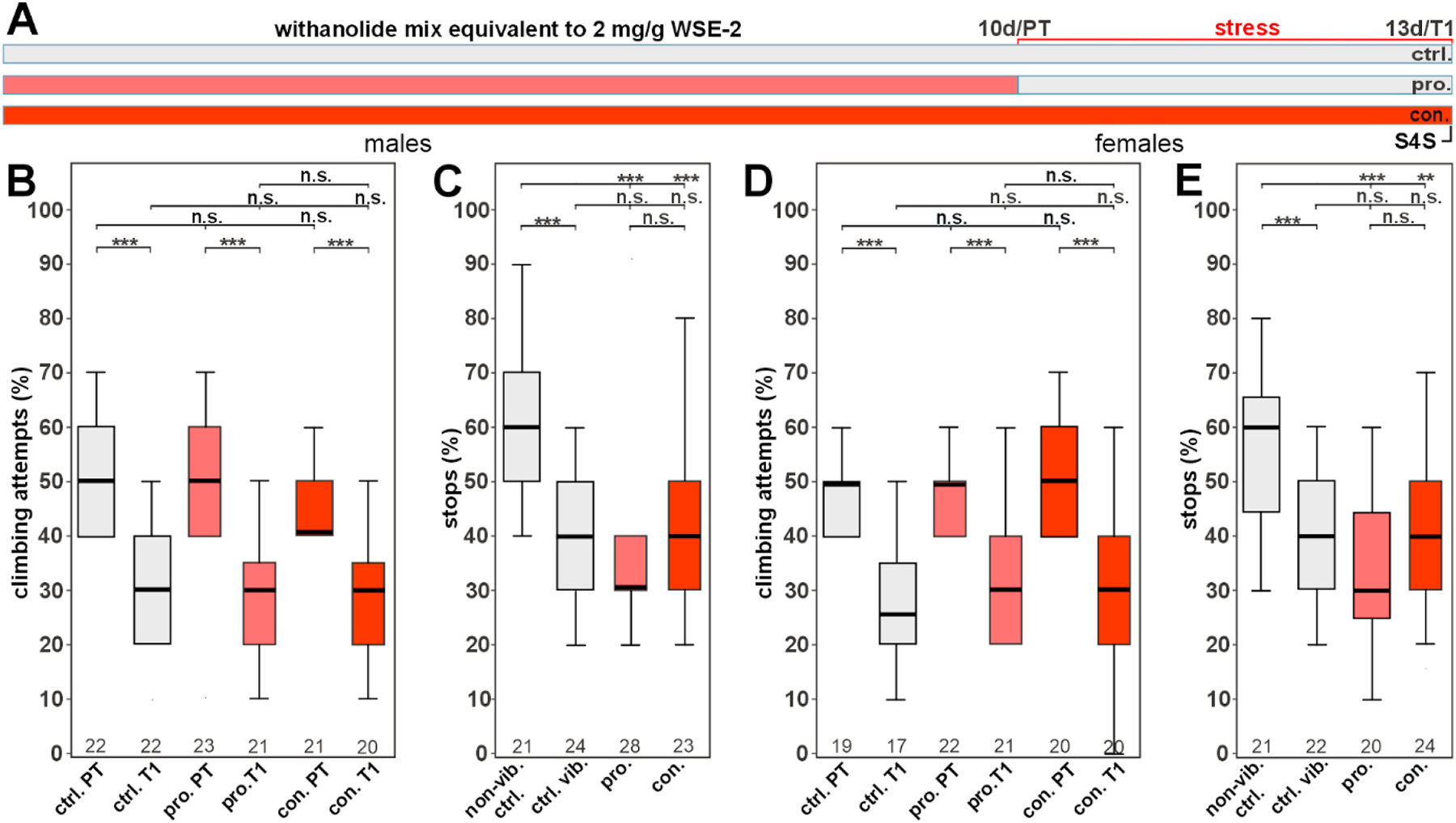
A withanolide mix does not improve stress-induced behavioral changes (A) Treatment outline schematic: the time of treatment with the withanolide mix equivalent to 2 mg/g WSE-2 is shown in red, time of food administration without the mix is shown in gray. (B) Percentage of gap-climbing attempts of males before (PT) and after (T1) stress exposure. At both timepoints, treated males (light red = prophylactic treatment, dark red = continuous treatment) perform similarly to the controls (gray). (C) Percentage of stops of males at a sweet-tasting stripe. All the stressed males perform significantly worse than the unstressed control (non-vib.), and the treated flies are not different from the untreated control. Similarly, treatment of females did not result in an improvement in gap-climbing (D) or in the S4S test (E). A pairwise Wilcoxon test with Bonferroni–Holm correction for multiple comparisons was used in each panel. The number of analyzed flies is given below the boxes. The horizontal bars in the box plots represent the medians, boxes the 25 % and 75 % quartiles, and whiskers the data points within ±1.5 times the interquartile range (IQR). Abbreviations: S4S = stop-for-sweets test, ctrl. = control, vib. ctrl. = vibrated control, non-vib. ctrl. = non-vibrated control, pro. = prophylactic treatment, con. = continuous treatment. **p < 0.01, ***p < 0.001, n. s. = not significant.

We previously showed that both an aqueous (WSAq) and a hydroethanolic (WSE) extract of WS root can promote resilience to a stress-induced depression-like state (DLS) in *Drosophila* (Holvoet et al., 2022). Stress was induced in 12–13d-old flies by phases of vibrations over a period of three days, after which the flies were tested for their motivation to climb an insurmountable gap and for stopping for sweet (S4S) as a test for anhedonia. We found that the aqueous WSAq extract was more potent, improving these stress-induced behaviors already at a concentration of 0.5 mg/g whereas WSE was only protective at 2 mg/g. Our analytical measurements found that the levels of the analyzed withanolides were increased between three- and ten-fold in WSE compared to WSAq extracts (Holvoet et al., 2022). This suggested that the withanolides did not mediate the resilience-promoting effect on the stress-induced DLS. To confirm this, in the present study, we tested whether a mix of these withanolides at a concentration corresponding to their level in 2.0 mg/g WSE-2 had a beneficial effect, using three treatment paradigms. As shown in Fig. 2 A, flies either received the withanolide mix only before the stress was applied (prophylactic treatment or pro.) or they were kept on the supplemented food for the duration of the experiment (continuous treatment or con.). Control flies received standard food (ctrl.). Neither the prophylactic nor the continuous treatment increased the gap climbing attempts in stressed males or females and the treated flies were indistinguishable from the treated controls (Fig. 2 B, D at T1). Unstressed treated flies also performed equally well as the controls, showing that the treatment neither had a positive nor a negative effect (Fig. 2 B, D at PT). Furthermore, all the stressed flies performed significantly worse than unstressed flies. Using the S4S test showed a similar outcome with neither prophylactic nor continuous treatment improving the behavior (Fig. 2C and E). This confirms that the tested withanolides do not provide resilience against stress-induced behavioral changes. In an additional experiment, mixing the withanolides with the alkaloid tropine at their concentrations present in WSE-2, 2 mg/mL, we found a significant improvement in the S4S assay in males. Continuously treated stressed males were not significantly different in the gap-climbing assay when compared before and after the stress was applied (Fig. S5).

### 4.4. A polar subfraction of WS root aqueous extract provides resilience to stress-induced behavioral changes in Drosophila

**Fig. 3.**
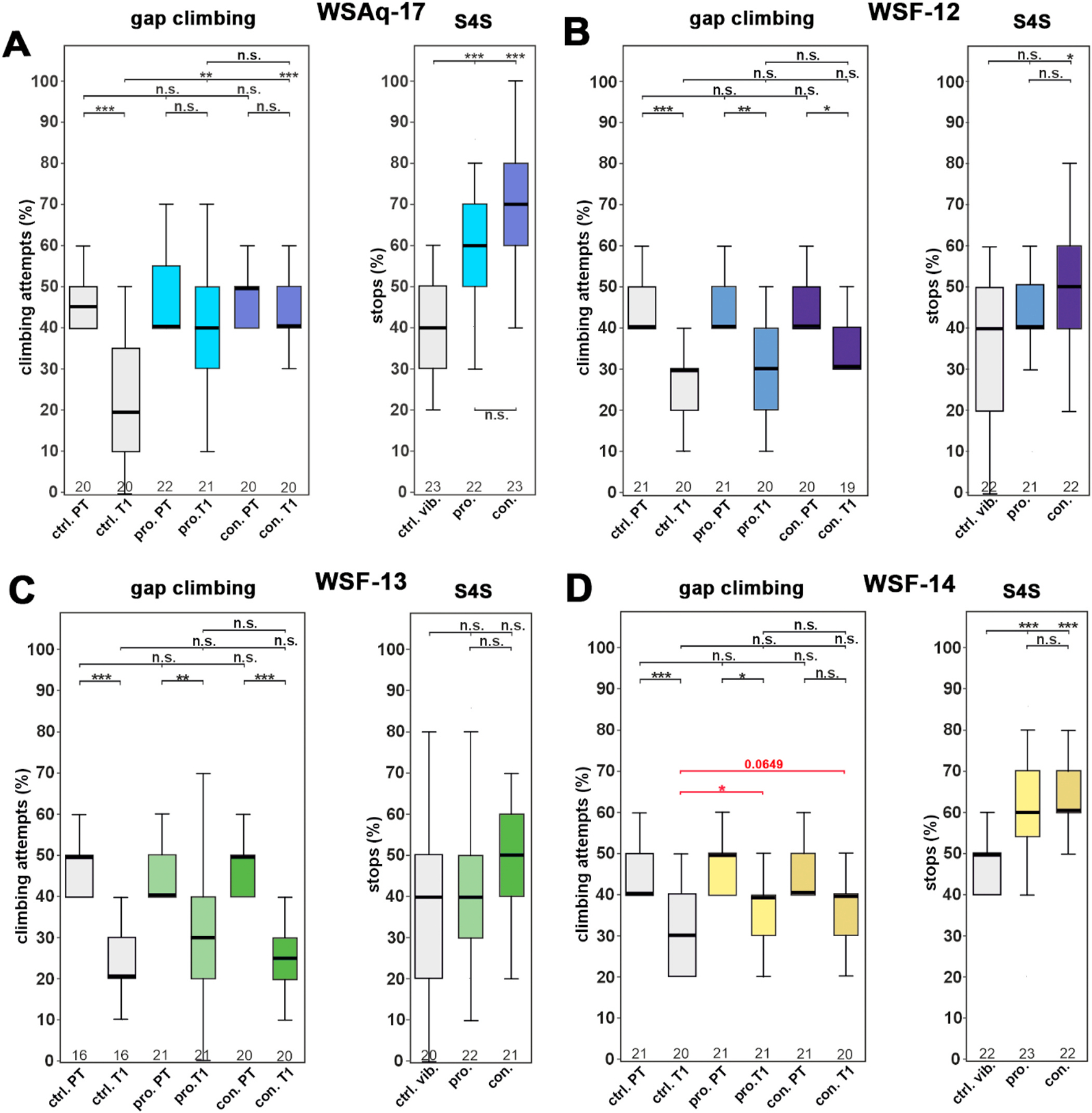
The WSF-14 polar subfraction derived from WSAq-17 protects against vibrational stress. (A) Treatment with WSAq-17 at 0.5 mg/g restores gap-climbing (left panel) and stop-for-sweet (S4S; right panel) with both treatment protocols. (B) Treatment with the WSF-12 subfraction at 0.5 mg/g had no protective effect except for a small improvement in S4S when using continuous supplementation. (C) The WSF-13 subfraction, given at 1 μg/g, did not improve either of the stress-induced behavioral changes. (D) When the flies were treated with WSF-14 at 7 μg/g, continuously treated stressed flies were not significantly different from their untreated controls. The difference in prophylactically treated flies was smaller than in the control flies but they still performed significantly worse than their unstressed control. Neither treatment resulted in a significant improvement of stressed treated flies versus stressed untreated controls when using a pairwise Wilcoxon test with Bonferroni–Holm correction for multiple comparisons. However, the prophylactically treated flies were significantly different from the untreated ones in a pairwise comparison without Bonferroni-Holm correction (red lines and asterisks). Only Wilcoxon tests with Bonferroni–Holm correction for multiple comparisons (black lines and asterisks) were used in the other panels. The number of analyzed flies is given below the boxes. The horizontal bars in the box plots represent the medians, boxes the 25 % and 75 % quartiles, and whiskers the data points within ±1.5 times the interquartile range (IQR). Abbreviations: PT = pre-stress, T1 = post-stress, ctrl. = control, vib. ctrl. = vibrated control, non-vib. ctrl. = non-vibrated control, pro. = prophylactic treatment, con. = continuous treatment. *p < 0.05, **p < 0.01, ***p < 0.001, n.s. = not significant.

Towards identifying active compounds that protect against the stress-induced DLS, we fractionated of a new batch of WS root aqueous extract (WSAq-17). As shown in Fig. S3A this resulted in an ethanol insoluble fraction (WSF-12), and a non-polar (WSF-13) and a polar (WSF-14) fraction. We then used these subfractions along with the parent WSAq-17 extract for the same feeding protocol as described for the withanolide mix (see Fig. 3A). We only tested males because our previous experiments with WSAq-9 showed very similar induction of resilience against vibrational stress for both sexes. As expected, WSAq-17 improved the gap-climbing and stop-for-sweet behavior of stressed flies when treated prophylactically or continuously compared to untreated controls (Fig. 3A). In addition, the performance of stressed flies was not significantly different from non-stressed flies when treated whereas the control flies showed the expected reduction in performance in these assays when stressed. When feeding WSF-12, at a concentration of 0.5 mg/g similar to its percentage of the starting material (97 %), we did not detect a protective effect on gap climbing but their anhedonia in the stop-for-sweet assay was slightly improved when fed continuously (Fig. 3 B). Similarly, treatment with WSF-13 (at a concentration of 1 μg/g) had no positive effect and all the stressed flies performed worse in the gap climbing assay than in the pre-test before they were vibrated (Fig. 3C). There was also no increase in stopping at a sweet-tasting stripe in the treated flies. When using WSF-14 (at 7 μg/g), we observed an increase in climbing attempts in the treated flies and while the stressed, prophylactically treated flies still performed worse at T1 than in the pre-test, the continuously treated flies were not significantly different at T1 than in the pre-test (Fig. 3 D). Although the improvement was not significantly increased when comparing the stressed treated flies to the untreated controls when performing a multiple comparison, this did reach significance with a direct pairwise comparison between the control and the flies treated prophylactically (for the continuously treated flies the p-value in the pairwise comparison was 0.0649). In the stop-for-sweet assay, both treatments resulted in significant improvement.

### 4.5. Withanolides were not detected in bioactive subfractions of WS aqueous extracts, whereas alkaloidal compounds are present

**Fig. 4.**
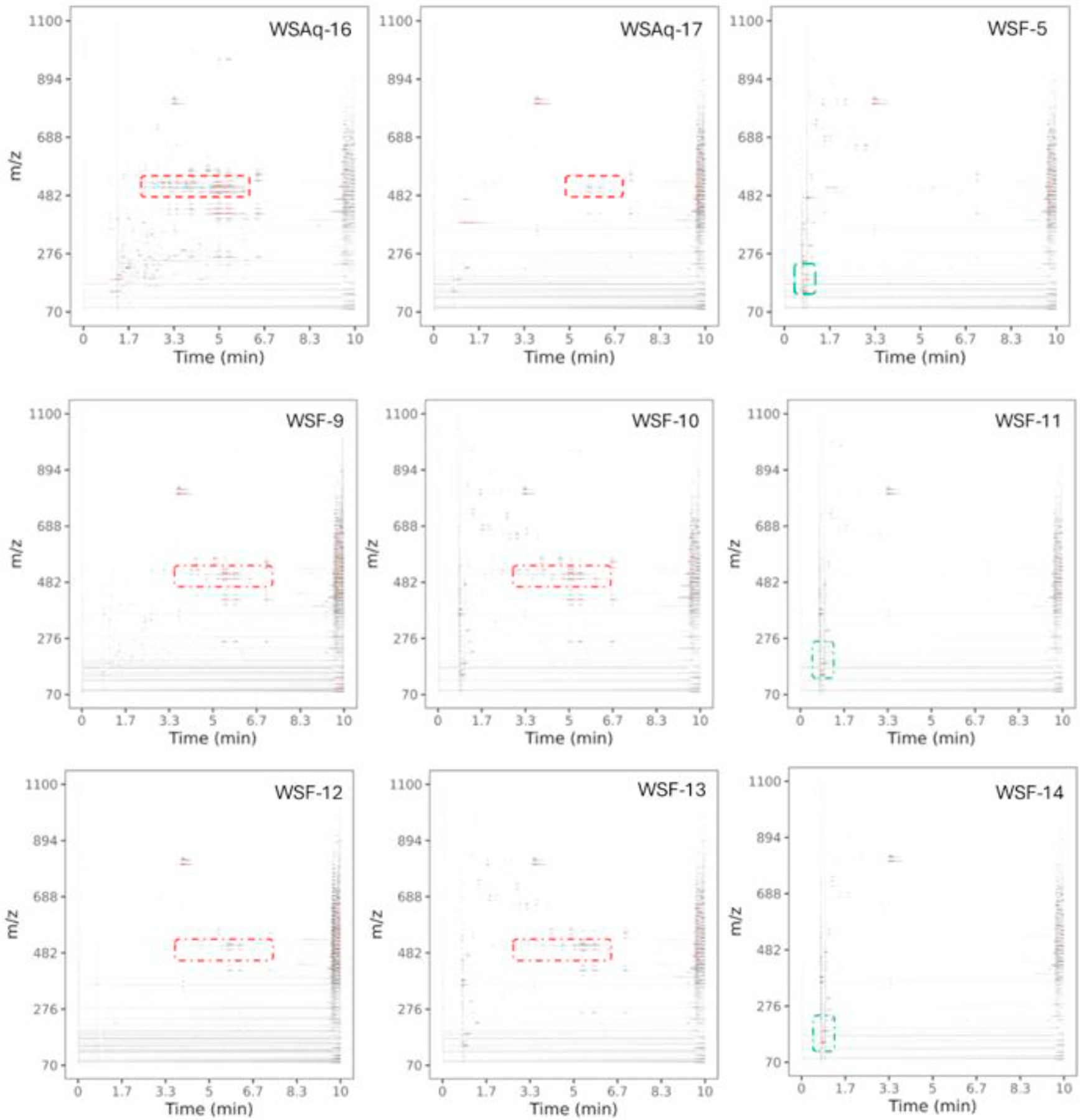
2D chromatographic and mass spectral representations of untargeted data collected by UPLC qTOF mass spectrometry. Plots show only signal from MS1 scans. The region around m/z 471–493 and 4–8 min shows the ion signals of withanolides (red region), as verified by authentic standards. Polar fractions (WSF-11 and WSF-14) notably do not contain withanolides but show bioactivity in the primary neuron and Drosophila assays. In this phenyl-3 separation, tropane alkaloids are barely retained and elute between 0 and 2 min (green region). Tropane alkaloids were also observed in the bioactive, polar subfractions (WSF-11 and WSF-14). As expected tropane alkoids were detected in the alkaloidal fraction (WSF-5).

Untargeted LC-HRMS/MS analysis was used to explore the chemical composition of the fractions obtained from the aqueous extracts WSAq-16 and WSAq-17, their fractions WSF-9 to WSF-14 and the alkaloidal extract WSF-5. The composition of each of these samples is shown as a 2-D ion map in Fig. 4. The data showed the presence of withanolides in the parent aqueous extracts WSAq-16 and WSAq-17. Withanolides were also present in fractions WSF-9 and WSF-10 which were inactive in the primary neuron assay but were not detected in the polar fraction, WSF-11, which was active in promoting dendritic complexity. Similarly fractions WSF-12 and WSF-13 contained withanolides but were not active in the *Drosophila* stress assays, whereas the polar fraction WSF-14 which was active in these assays did not have detectable levels of withanolides. More detailed analysis of fractions WSF-12, 13 and 14 by Global Natural Products Social Molecular Networking (GNPS) analysis is provided in supplemental material (Fig. S3D).

### 4.6. Tropane alkaloids are present in active WS extracts and fractions

**Fig. 5.**
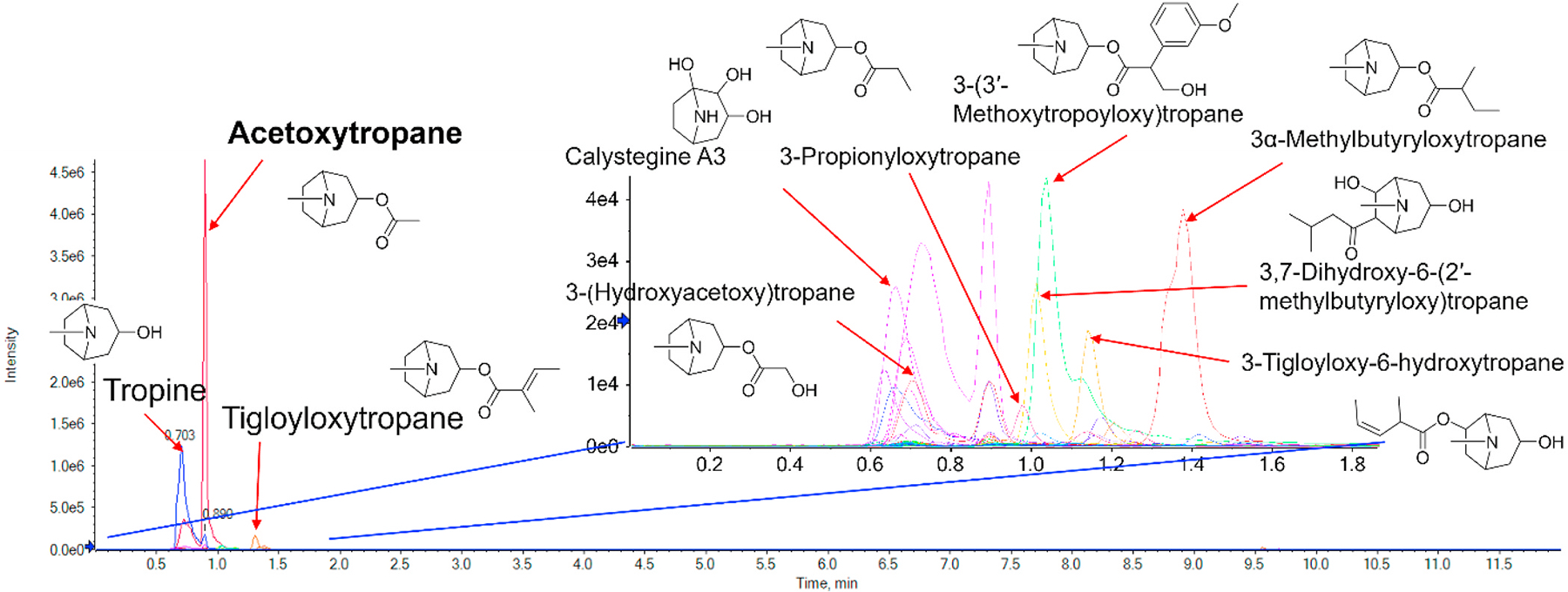
LC-HRMS/MS analysis suggests the presence of tropane alkaloids in the active polar fraction WSF-14. Tentative level 1 annotations (L1 annotations) were obtained by matching the MS1 ion signals to a library of accurate monoisotopic masses of known tropane alkaloids (Table S5). Where possible (i.e. data-dependent acquisition acquired MS2 fragmentation), MS2 fragmentation data was inspected to verify characteristic fragmentation of the tropane ring.

LC-HRMS/MS analysis of the active fraction WSF-14 suggested the presence of tropane alkaloids in this sample based on MS1 matching and GNPS molecular networking based on MS/MS spectral similarity (Fig. 5 and Fig. S3). HRMS data of peaks and their annotation levels given in Table S5. The most prominent peak (retention time 0.89 min) was tentatively assigned to acetoxytropane (Fig. 5). However, sole mass spectrometry-based methods are unable to determine positional isomers (acetyl group in 1, 2 or 3-position) nor the possible stereoisomers (α-isomer, substituent below the plan of the ring, or β-isomer, substituent oriented above the plane of the ring). The subfamily Solanoideae within the Solanaceae family contain a large range of tropane alkaloids and their biosynthesis pathway yields 3-acetoxytropanes with α- or β-configuration dependent on the presence of tropine reductase 1 (TR I) or tropine reductase 2 (TR II), respectively (Portsteffen et al., 1994) Acyltransferases specific to tropine (tropan-3α-ol) and pseudotropine (tropan-3β-ol) have been differentiated in other Solanaceous species (Robins et al., 1991) catalyzing the formation of 3α- and 3β-forms of 3-acetoxytropane (Robins et al., 1991).

We therefore synthesized both stereoisomers of 3-acetoxytropane (Supplemental Section S1). Both products were observed by mass spectrometry at the expected m/z of 184 ([M+H]+. Initial 1D NMR gave the following results: 3α-acetyloxytropane (acetyltropine) HCl. 1H NMR (chloroform-d) δ 12.55 (s, 1H); 5.14 (t, 1H) 3.75 (s, 2H); 3.10 (d, 2H); 2.75 (s, 3H); 2.40 (d, 2H); 2.24 (d, 2H); 2.08 (s, 3H); 2.00 (d, 2H). 3β-acetyloxytropane (acetyl exo-tropine) HCl. 65 mg (85 % yield). 1H NMR (chloroform-d) δ 142.84 (s, 1H); 5.06 (m, 1H); 3.80 (s, 2H); 2.74 (m, 5H); 2.27 (m, 2H); 2.13 (m, 4H); 2.05 (s, 3H). Two-dimensional NMR (Fig. S10) verified the stereochemistry of the two isomers as a 3α-acetyloxytropane (acetyltropine) and 3β-acetyloxytropane (acetyl exotropine).

### 4.7. Further exploration of the role of alkaloids in the bioactivity of WS

#### 4.7.1. An alkaloidal extract of WS root enhances resilience to stress in Drosophila

**Fig. 6.**
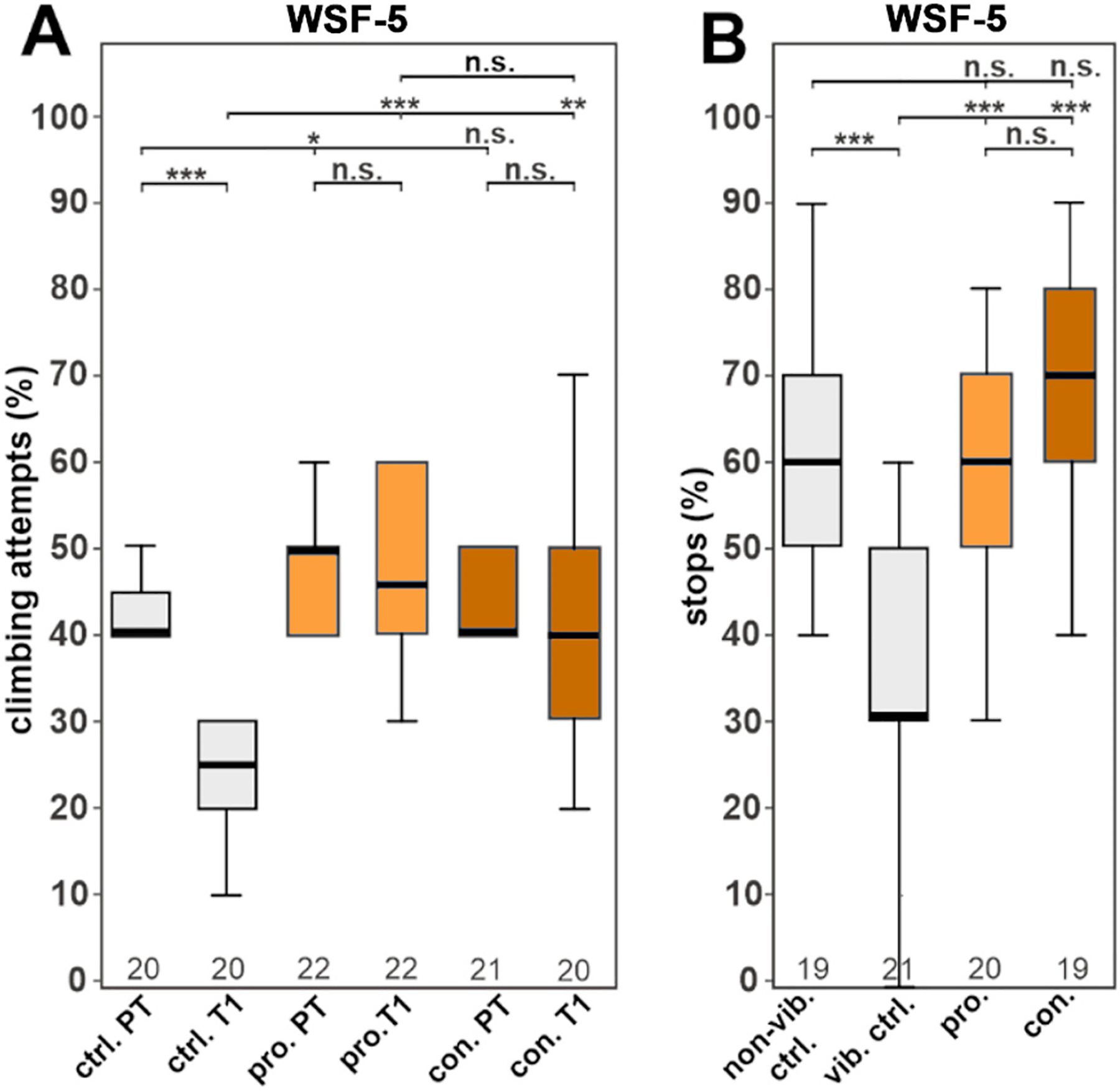
The alkaloid-containing WSF-5 subfraction provides resilience to chronic stress in *Drosophila* (A) Treatment with WSF-5 at 10 μg/g increases gap-climbing attempts after stress exposure when given prophylactically or continuously. (B) Both treatment protocols also increase the percentage of stops at a sweet-tasting stripe. All flies were males. A pairwise Wilcoxon test with Bonferroni–Holm correction for multiple comparisons was used in each panel. The number of analyzed flies is given below the boxes. The horizontal bars in the box plots represent the medians, boxes the 25 % and 75 % quartiles, and whiskers the data points within ±1.5 times the interquartile range (IQR). Abbreviations: PT = pre-stress, T1 = post-stress, ctrl. = control, vib. ctrl. = vibrated control, non-vib. ctrl. = non-vibrated control, pro. = prophylactic treatment, con. = continuous treatment. *p < 0.05, **p < 0.01, ***p < 0.001, n.s. = not significant.

To verify the role of alkaloids in the effects of WS on stress, we tested WSF-5, which was prepared by a standard alkaloidal extraction protocol (Houghton and Raman, 1998) involving extracting the alkaloids as ionized salts using acid, and removal of non-polar unionized materials by extracting with methylene chloride, then converting the salts back to bases by addition of alkali and extraction of the bases into methylene chloride (Fig. S4). As determined by LC-HRMS/MS analysis, this subfraction was rich in alkaloids, including tropane alkaloids and contained acetyltropine (Fig. 5). WSF-5 was tested at a concentration (10 μg/g food) based on its weight as a percentage of the starting BEN-WS-8 root material. Treatment with WSF-5 completely prevented the adverse effects of the vibration on both gap climbing and S4S behaviors and the stressed flies performed as well as the flies that were not stressed in the gap-climbing assay (Fig. 6A and B). Furthermore, both prophylactic and continuous treatment improved the behavior of stressed flies when compared to the stressed controls.

#### 4.7.2. The acetoxytropane isomers, acetyltropine and acetyl exotropine, can be separated chromatographically; acetyltropine is the only isomer detected in BEN-WS-8 and its preparations

**Fig. 7.**
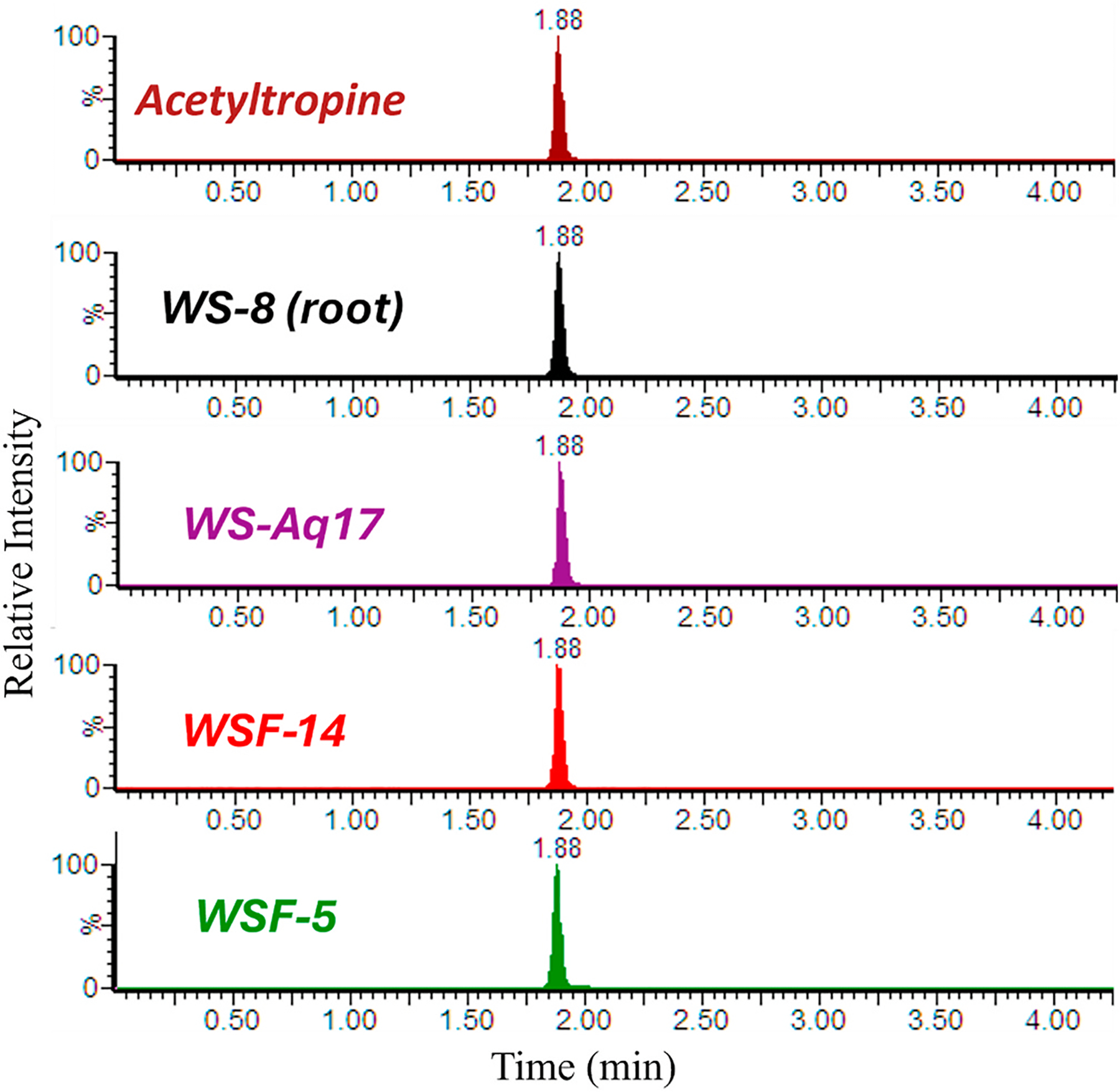
Acetyltropine, (but not the isomer acetyl exotropine) was detected in the WS root sample, and bioactive extracts and fractions derived from it. Acetyltropine (α-isomer)and acetyl exo-tropine (β isomer) were clearly distinguishable by two-dimensional NMR (Fig. S10) and can be resolved chromatographically using the developed C18 method in conjunction with mass spectrometry (section 3.5.1) (Fig. S11). Acetyltropine was detected in the original WS root sample, BEN-WS-8 (WS-8), its aqueous extract (WSAq-17), the polar fraction of WSAq-17 (WSF-14) and an alkaloidal extract (WSF-5) of the same root sample.

Using the optimized LC-C18 method (supplementary section S2), acetyltropine and acetyl exo-tropine eluted with distinct retention times at close to 1.85 and 1.45 min, respectively (Fig. S11). A spike-in experiment into the LC-MRM-MS sample extract (section 3.6.2) of WS-8 root indicated that the 3α-isomer acetyltropine is the predominant species in the WS-8 root extract. Acetyl exotropine (3β isomer) was not detectable in the WS-8 root extract under the applied conditions. LC-MRM-MS analysis of the aqueous WS extract (WSAq-17), the active fraction WSF-14, as well as the alkaloidal fraction WSF-5 all showed LC-MRM-MS signal traces that confirmed the presence of acetyltropine, i.e. the α-isomer, with retention time 1.88 min (Fig. 7).

#### 4.7.3. Acetyltropine enhances resilience to stress in Drosophila

**Fig. 8.**
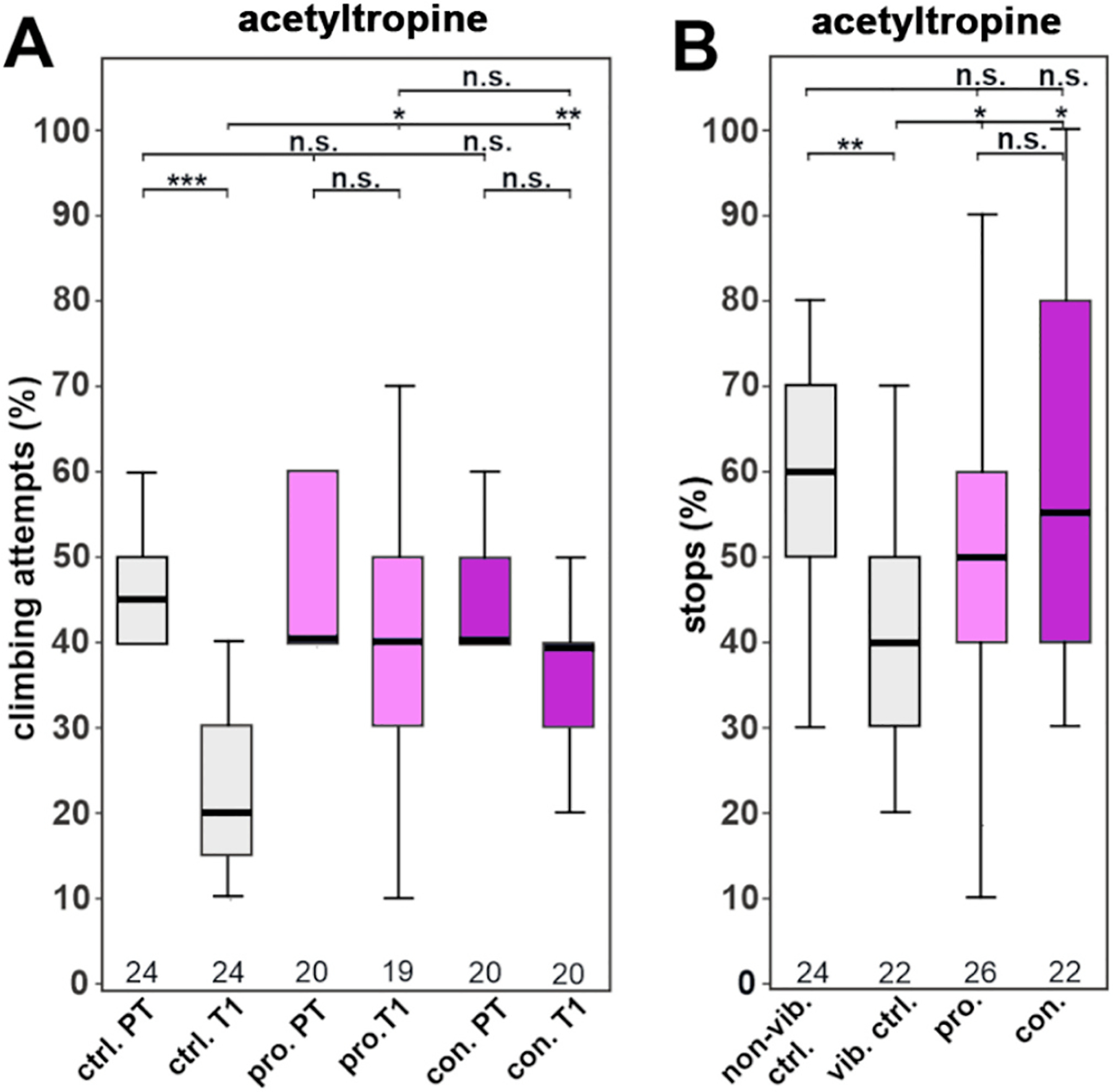
Acetyltropine supplementation (0.4 μg/g food) prevents stress-induced behavioral changes (A). The percentage of gap-climbing attempts after stress exposure is restored to the levels of unstressed flies after prophylactic or continuous treatment with acetyltropine. (B) The stops at a sweet-tasting stripe are increased in flies kept on acetyltropine-supplemented food either prophylactically or continuously. All flies were males. A pairwise Wilcoxon test with Bonferroni–Holm correction for multiple comparisons was used in each panel. The number of analyzed flies is given below the boxes. The horizontal bars in the box plots represent the medians, boxes the 25 % and 75 % quartiles, and whiskers the data points within ±1.5 times the interquartile range (IQR). Abbreviations: PT = pre-stress, T1 = post-stress, ctrl. = control, vib. ctrl. = vibrated control, non-vib. ctrl. = non-vibrated control, pro. = prophylactic treatment, con. = continuous treatment. *p < 0.05, **p < 0.01, ***p < 0.001, n.s. = not significant.

As described above, WSF-14 and WSF-5 contain a tropine alkaloid with the characteristic of acetyltropine which we had synthesized. We therefore tested whether the acetyltropine can provide resilience to the vibrational stress. As shown in Fig. 8, treating the flies with acetyltropine (0.4 μg/g), increased their gap-climbing and stopping at the sweet stripe. This was the case for prophylactic and continuous treatment, as both restored the behavior to the levels of unstressed flies.

#### 4.7.4. Acetyltropine levels varied between the extracts and fractions tested

Acetyltropine content was determined in the various extracts and fractions tested in the two bioassays (Table 1) using the LC-MRM-MS method described earlier (section 3.5.1). Validation of the method for quantitation of acetyltropine is described in supplementary section S2.2. The concentration of acetyltropine in the primary neuron cell culture media and in the *Drosophila* food containing each of the extracts or fractions could then be calculated and compared (Table 1).

The bioactive alkaloidal fraction (WSF-5), the two aqueous extracts (WSAq-16 and WSAq-17) and their active, polar fractions (WSF-11 and WSF-14 respectively) all contained acetyltropine. Acetyltropine was present at much lower levels in the inactive non-polar fractions WSF-10 and WSF-13. These data are consistent with the hypothesis that acetyltropine is one of the bioactive compounds responsible for enhanced dendritic arborization in the primary neurons and reduced stress-related behavioral changes in *Drosophila*. The two ethanol-insoluble fractions (WSF-9 and WSF-12) were both inactive in the bioassays, but surprisingly contained similar levels of acetyltropine as the bioactive parent extracts (WSAq-16 and WSAq-17). This suggests that other components of WSF-9 and WSF-10 may inhibit the activity of acetyltropine or possess other anti-synergistic activities. Four other peaks observed in LC-HRMS/MS of the extracts and fractions tested in bioassays were assigned as the tropane alkaloids tropine, 3-tigloyloxytropane, 2,7-dihydroxy-6-(2′-methylbutyryloxy)tropane and 3-hydroxyacetoxytropane based on their high resolution mass spectra. Their relative peak areas in the WS extracts and fractions tested in the bioassays, as well as their HRMS data are provided in Figs. S8 and S9.

## 5. Discussion

The Ayurvedic rejuvenating herb ashwagandha (*Withania somnifera*; WS) is one of the most popular botanical supplements available on the market today. As a dietary supplement it is used predominantly as an adaptogen to improve stress management, sleep, and mental and physical endurance (Sprengel et al., 2025). The widespread use of WS highlights the need for rigorous research to support and inform its evidence-based use. The biological activity of botanical products can be optimized by ensuring that they contain the relevant active compounds at the doses required for activity. Despite extensive literature on WS (Mikulska et al., 2023) direct associations between its various phytochemical components and its many functional benefits remain to be demonstrated.

In an earlier study we compared the biological effects and chemical profile of WS root aqueous (WSAq) and hydroethanolic (WSE) extracts (Holvoet et al., 2022); both types of extract are frequently used in WS botanical supplements including in products that have shown effects on cognition, stress and sleep in human clinical trials (Ng et al., 2020; Speers et al., 2021). We started with the expectation that WSE would have stronger biological activity due to its higher content of withanolides. This group of compounds, comprising up to 1.5 % by weight of dry WS, has commonly been credited with eliciting many of the biological effects of the herb (Singirala et al., 2025). However, in a *Drosophila* model, higher doses of WSE extract than WSAq extract were required to elicit resilience to stress-induced behavioral changes (Holvoet et al., 2022), despite higher withanolide content in WSE than WSAq extracts.

These results were echoed in the present study (Fig. 1A), where an aqueous WS root extract (WSAq-9, 100 μg/mL) enhanced dendritic complexity in mouse primary hippocampal neurons while a hydroethanolic extract (WSE-2, 100 μg/mL) inhibited dendritic complexity compared to controls, while having no effect at a lower dose (50 μg/mL). Furthermore, a mixture of withanolides equivalent to their content in the active extracts did not increase dendritic arborization in primary neurons (Fig. 1A) or provide resilience to stress in the *Drosophila* model (Fig. 2).

Withanolides have previously been reported to affect neuronal axonal or dendritic growth. Withanolide A, withanoside IV and withanoside VI stimulated neurite outgrowth in SH-SY5Y cells (Kuboyama et al., 2002). Withanolide A, withanoside IV, and its aglycone sominone stimulated axonal and dendritic regeneration and synaptic reconstruction following β-amyloid-induced atrophy in rat cortical primary neuron cultures (Kuboyama, Tohda and Komatsu, 2005, 2006). Oral administration of withanoside IV also attenuated β-amyloid-induced axonal, dendritic and synaptic losses in mice, however only its aglycone sominone was detected in plasma (Kuboyama et al., 2006). These data indicated that withanolides can also improve neuroplasticity in primary neurons following β-amyloid exposure. The lack of effect of the withanolide mixture (WSAq-9 mix) in our healthy primary neuronal culture may reflect the need for an insult causing synaptic impairments to be present to observe the protective effects of the withanolides. It may also have been due to the lower concentration of withanolides tested in our primary neuron assay (withanolide A, 0.21 μM; withanoside IV 0.03 μM; total withanolides 0.53 μM) compared to the *in vitro* studies listed above (all at 1 μM).

The apparent lack of involvement of withanolides in the two bioassays examined led to a further exploration of the other possible active compounds in WS. Fractionation of active WSAq extracts followed by their biological testing and LC-HRMS/MS analysis demonstrated that, in both the *Drosophila* and primary neuron models, biological activity was retained in polar fractions (Figs. 1B and 3) that did not contain detectable withanolides, but were rich in tropane alkaloids (Fig. 4, S3). An alkaloidal extract of WS root (WSF-5) also ameliorated stress induced behavioral changes in *Drosophila* (Fig. 6).

The compound acetoxytropane was observed to be the most abundant alkaloid in the active fraction WSF-14 (Fig. 5). Since this compound can exist in two isomeric forms acetyltropine (3α-acetyltropine) and acetyl exotropine (3β-acetyltropine) a LC-MRM-MS method was developed to resolve these isomers (supplemental section S2). Of the two isomers, only acetyltropine was detected in the bioactive extracts and fractions (Fig. 7). Acetyltropine also ameliorated stress-related behavioral effects in *Drosophila* (Fig. 8) when administered at a dose similar to that delivered by the water extract WSAq-17, clearly substantiating its role in the bioactivity of WS.

It was notable that the amount of acetyltropine delivered by the different bioactive WS extracts and fractions varied widely (Table 1). Conversely, fractions WSF-9 and WSF-12 had no, or lower activity compared to the parent water extracts (WSAq-16 and WSAq-17) but contained similar or greater levels of acetyltropine. These results indicate the modulatory effects of additional compounds present in these fractions that could exert additive, synergistic or antagonistic effects to acetyltropine in the bioassays. The nature of these compounds and the type of interaction with acetyltropine requires further investigation.

Notably in this study, we combined chemical profiling of extracts and fractions using mass spectrometry with bioactivity studies. Liquid chromatography in conjunction with high resolution tandem mass spectrometry (LC-HRMS/MS) has proven valuable in the phytochemical analysis of botanical extracts including ashwagandha (Marney et al., 2024; Ha et al., 2022; Abraham and Kellogg, 2021). Chemical profiling by mass spectrometry (metabolomics) provides comprehensive information on metabolites present in different parts of the plant and allows the defining of chemo-types dependent on geographical locations and growth stages.

To expand the understanding of WS’s neuroactive potential and active compounds, two distinct, medium throughput investigative approaches were employed, mouse primary neuron cultures and *Drosophila* bioassays. We observed an increase in dendritic growth and complexity in the primary neurons, both of which are key features associated with neuroplasticity in response to treatment with WS extracts and fractions. We also tested the potential protective effects of these test preparations in a *Drosophila* model of chronic stress induced by 3 days of repeated vibration episodes (Ries et al., 2017). While the assays and species used are distinct, the two biological effects examined in this study, stress-induced depression and neuronal plasticity, are related (Bennett and Lagopoulos, 2014, 2019). Chronic stress has been shown to result in reduced dendritic spine growth in the hippocampus, an effect believed to be mediated by brain derived neurotropic factor (BDNF). Reduced hippocampal dendritic arborization (complexity) can also result from chronic stress and accompanies depressive-like symptoms in mice (Hu et al., 2022; Gilabert-Juan et al., 2017). Therefore, measurement of dendritic complexity in cultured neurons may provide insight into possible anti-depressant effects. For example, an alkaloidal extract from *Sophora alopecuroides* L. increased BDNF secretion and dendritic complexity of mouse primary cortical neurons *in vitro*, and also increased dendritic complexity in the prefrontal cortex and ameliorated stress-induced depression of a mouse model *in vivo* (Li et al., 2023).

There is currently interest in developing 3-acyltropine derivatives as psychoplastogenic drugs; several, including scopolamine were able to increase dendritic spinogenesis in cortical neurons in culture (Chow et al., 2023). Scopolamine has been shown to decrease depression-like behavior in rats and increase spine density and maturation in rat prefrontal cortex brain slices (Voleti et al., 2013). Low doses of scopolamine showed antidepressant effects in 7 out of 8 human clinical trials (Kolar, 2021).

Similar anti-depressant effects and mechanisms involving improved neuroplasticity may be occurring with the tropane alkaloids such as acetyltropine found in WS. This work confirms the relevance of alkaloids as biologically active constituents of WS and emphasizes acetyltropine as a major and bioactive alkaloid in root of WS grown in Central Oregon. Ongoing research is required to confirm the effects of acetyltropine and related alkaloids found in WS on neuronal plasticity and depression, their bioavailability, and the optimum doses required to achieve these effects in humans.

Alkaloids are common in the Solanaceae family to which WS belongs, ranging from 0.13 % to 0.31 % of the dried WS plant material (Singirala et al., 2025). Most recent reviews list alkaloids among bioactive constituents of WS (Melzig, 2025; Arshad et al., 2025; Khalid et al., 2025; Pushpakaran et al., 2025). However, the few studies that were found in the literature directly investigating the biological activities of WS alkaloidal fractions, were performed in the 1960s (Prasad and Malhotra, 1968; Malhotra et al., 1965; Malhotra et al., 1965). These investigations examined the *in vivo* pharmacological effects of a chemically uncharacterized alkaloidal extract termed “ashwagandholine” and its fractions. The dearth of more recent studies specifically exploring the role of alkaloids in the functional effects of WS is surprising, although this may be due to the many independent studies on bioactivity of the same alkaloids found in other botanical sources. Further work consolidating the role of alkaloids in the beneficial effects of WS would justify the development of botanical products standardized to alkaloids as well as withanolides.

Potential interactions between the different compounds classes found in WS to achieve the observed effects warrants further investigation. For example, although the withanolide mixtures and withanolide rich fractions were inactive when tested in the present bioassays, it would be interesting to explore their possible additive, synergistic or antagonistic interactions with the alkaloids by testing combinations of the withanolide- and alkaloid-rich fractions. Acetyltropine could also be tested, at an active concentration, combined with the inactive fractions to similarly examine such interactions.

Several additional questions arise when considering the relevance of the present study’s findings to medicinal products or dietary supplements containing WS. For example, are alkaloids like acetyltropine likely to be stable during processing of WS, and therefore present in these products? We did not specifically address stability of the tropane alkaloids in this study. However, the alkaloids were present in extracts prepared by boiling plant material in water or dilute acid at 100 °C for 1.5 h. Acetyltropine also remained detectable in root extracts that had been stored in the freezer (−20 °C) for at least three years. While some degradation is certainly possible, we did not see any problems as compared with other more unstable compounds (i.e. dramatic increases in variability over time in our quality control Levy-Jennings plots (Fig. S12). Studies reported in the literature show that the tropane alkaloids atropine and scopolamine are able to withstand the temperatures involved in baking (180 °C) (González-Gómez et al., 2024). However, instability at much higher temperatures, such as in a GC-MS inlet at 275 °C, has been noted for these two alkaloids (Koželj and Prosen, 2021). This data suggests that tropane alkaloids from WS are likely to be present in dietary supplements and to remain stable under normal storage conditions. Future studies could examine the levels and stability of acetyltropine and related bioactive alkaloids in commercially available dietary supplements using the methods described in the present study.

The oral bioavailabilty of acetyltropine and its ability to cross the blood brain barrier (BBB) is another important consideration which does not appear to have been evaluated to date for this compound. However, classical tropane alkaloids such as atropine, scopolamine and cocaine are well established to penetrate the central nervous system (CNS), which underlies both their therapeutic and toxic central effects (Cornelissen et al., 2020; Shim et al., 2022). By contrast, more polar nortropane derivatives, including calystegines, show limited or negligible BBB permeability and therefore lack CNS activity (Shim et al., 2022). These findings highlight that tropane alkaloids encompass compounds with divergent BBB penetration profiles, and future work will be needed to directly evaluate acetyltropine’s pharmacokinetics and brain distribution in order to determine its translational potential.

Tropane alkaloids are already highly valued for their medical uses as antispasmodics, antiemetics, local anesthetics, bronchodilators and mydriatics, due primarily due to their anticholinergic effects (Shim et al., 2022; Wang et al., 2024). However, these same properties can lead to adverse effects through exposure to toxic levels in poisonous plants or contaminated foods (EFSA, 2013; Kwakye et al., 2018; Wang et al., 2024). Adverse anticholinergic effects have not been widely associated with the use of WS products as botanical supplements or in clinical trials (Tandon and Yadav, 2020). Further experiments assessing the effective dose of acetyltropine in rodent models could provide clues for corresponding human doses using interspecies scaling (Nair and Jacob, 2016), which would allow an evaluation of potential for toxicity. Efforts to enhance alkaloid levels in commercial WS products to optimize their beneficial effects would need to be balanced carefully against the increased risk of adverse effects.

In the present study, WS extracts provided resilience to stress-induced depression-like behavior in *Drosophila* flies, and increased dendritic complexity in mouse primary neurons *in vitro*. Neurodegeneration, including dendritic regression (Mattson and Arumugam, 2018) is known to occur during aging. An excessive stress response in the hypothalamic pituitary axis, often seen during aging, can also lead to hippocampal dendritic atrophy, resulting in memory impairments (Sampedro-Piquero et al., 2018). Depression affects about 10 % of adults over 65 years of age (Almeida, 2012) and alleviation of depression has been linked to cognitive benefits (Ainsworth et al., 2024). Thus, the effects of WS and acetyltropine on dendritic arborization, and resilience to stress and depression observed in the present study are consistent with the ethnopharmacological use of WS in Ayurvedic medicine as a rasayana or rejuvenating herb (Gautam and Pandey, 2020; Singh et al., 2011).

## 6. Conclusions

This study challenges the conventional view that withanolides are the main bioactive constituents of *Withania somnifera* (WS) by demonstrating that other compound classes - in this study alkaloids—play a role in mediating neuroprotective and stress-resilience effects. Using primary hippocampal neuron cultures, we observed enhanced dendritic complexity upon treatment with specific WS fractions lacking withanolides, indicating that non-withanolide constituents contribute to neuronal complexity. In parallel, *Drosophila* behavioral assays revealed that alkaloid-rich extracts and subfractions especially WSAq-17, WSF-5 and WSF-14, showed protective effects against a depression-like state in chronically stressed flies. Among the identified alkaloids, acetyltropine was quantified and confirmed as a major constituent, also capable of conferring resilience to stress in *Drosophila*. Collectively, our findings highlight the importance of previously underexplored alkaloidal fractions in WS, positioning acetyltropine as a promising candidate for further investigation.

## Supplementary Material

MMC2

MMC1

## Figures and Tables

**Table 1:** Acetyltropine content in WS root aqueous extracts (WSAq-16 and WSAq-17), their fractions (WSF-9 to WSF-14) and an alkaloidal fraction (WSF-5). The data was used to calculate acetyltropine concentration when each material was incorporated into primary neuron cell culture media or *Drosophila* food.

Test material	Acetyltropine (μg/mg) in test material^a^	Acetyltropine in cell culture medium (nM) or *Drosophila* food (ng/g)^b^	Bioactivity
Materials tested in the mouse primary hippocampal neuron bioassay:			
WSAq-16	0.23 ± 0.01	126 nM	Active
WSF-9	0.56 ± 0.02	292 nM	–
WSF-10	0.01 ± 0.0004	0.01 nM	–
WSF-11	1.04 ± 0.05	3.9 nM	Active
Materials tested in the *Drosophila* stress assay:			
WSAq-17	0.39 ± 0.008	195 ng/g	Active
WSF-12	0.23 ± 0.007	115 ng/g	Partially active
WSF-13	0.01 ± 0.002	0.01 ng/g	–
WSF-14	1.00 ± 0.1	7.00 ng/g	Active
WSF-5	0.09 ± 0.004	0.9 ng/g	Active
Acetyltropine	N/A	400 ng/g	Active

a Mean ± SD of three replicate determinations.

b Concentration of material tested × mean acetyltropine content.

## Data Availability

Data will be made available on request.

## Appendix A. Supplementary data

Supplementary data to this article can be found online at https://doi.org/10.1016/j.jep.2025.120905.

## List of abbreviations:

1D-NMR: one-dimensional nuclear magnetic resonance
2D-NMR: two-dimensional nuclear magnetic resonance
AALAC: Association for Assessment and Accreditation of Laboratory Animal Care
AHP: American Herbal Pharmacopoeia
ANOVA: analysis of variance
Anti-Anti: antibiotic-antimycotic
AraC: arabinofuranoside hydrochloride
AUC: area under the curve
BDNF: brain derived neurotropic factor
BEN: BENFRA Center
Con.: continuous treatment
CS: Canton-Special strain
Ctrl: contro
DIV: days *in vitro*
DLS: depression like state
ESI+: positive electrospray ionization
FA: formic acid
FBS: fetal bovine serum
GNPS: Global Natural Products Social Molecular Networking
IQR: interquartile range
LC: liquid chromatography
LC-HRMS/MS: liquid chromatography coupled to high resolution tandem mass spectrometry
LC-MRM-MS: liquid chromatography coupled to multiple reaction monitoring mass spectrometry
MEM: minimal essential medium
MRM: multiple reaction monitoring
MS: mass spectrometry
MS/MS: tandem mass spectrometry
NRM: nuclear magnetic resonance
Non.vib.: non-vibrated
n.s.: not significant
NT: not tested
OHSU: Oregon Health and Science University
OSU: Oregon State University
OWH: Oregon’s Wild Harvest
Pro.: prophylactic treatment
PT: pre-test
SEM: standard error of the mean
S4S: stop-for-sweet
T1: post-stress tests
TR: tropine reductase
TOF-MS: time-of-flight mass spectrometry
UPLC qTOF: ultra-high performance liquid chromatography coupled with quadrupole time-of-flight
Vib.: vibrated
WS: Withania somnifera
WSAq: *Withania somnifera* aqueous extract
WSE: *Withania somnifera* 70 % ethanol extract
WSF: *Withania somnifera* fraction
