# Supplementary material for: Mouse neuronal dendritic complexity and resilience to stress-induced depression in Drosophila melanogaster are enhanced by Withania somnifera alkaloids
Source: J Ethnopharmacol. Author manuscript; Available in PMC 2026 Feb 5. (PMC12875680; doi:10.1016/j.jep.2025.120905)
Supplement: MMC2 [file NIHMS2126075-supplement-MMC2.docx]

**SUPPLEMENTAL MATERIAL**

**Title:**

**Mouse neuronal dendritic complexity and resilience to stress-induced depression in *Drosophila melanogaster* are enhanced by *Withania somnifera* alkaloids.**

**Author list:**

Rudranil Dutta^a,b,^**^†^**, Helen Holvoet^c,^ **^†^**, Luke Marney^a,b,^ **^†^**, Kadine Cabey^a,d^, Cody Neff ^a,d^, Mikah Brandes^a,d^, Jonathan Zweig^a,d^, Jesus Martinez^a,d^, Jaewoo Choi^a,b^, Christine McClure ^a,d^, Md Nure Alam^a,b^, Liping Yang^a,b^, Burkhard Poeck^c^, Jan F. Stevens^a,e,f^ , Doris Kretzschmar^a,g^, Nora E. Gray^a,d^, Roland Strauss^c^, Claudia S. Maier^a,b,e^, Amala Soumyanath^a,d,*^

**Affiliations:**

1. BENFRA Botanical Dietary Supplements Research Center, Oregon Health & Science University, Portland, OR 97239, USA
2. Department of Chemistry, Oregon State University, Corvallis, OR, 97331, USA
3. Institute for Developmental Biology and Neurobiology, Johannes Gutenberg-Universität Mainz, Hanns-Dieter-Hüsch-Weg 15, 55128 Mainz, Germany
4. Department of Neurology, Oregon Health & Science University, Portland, OR 97239, USA
5. Linus Pauling Institute, Oregon State University, Corvallis, OR, 97331, USA
6. Department of Pharmaceutical Sciences, Oregon State University, Corvallis, OR, 97331, USA
7. Oregon Institute of Occupational Health Sciences, Oregon Health & Science University, Portland, OR 97239, USA

**†** These authors contributed equally to this work

*Correspondence to: Department of Neurology, Oregon Health & Science University, Portland, OR 97239, USA. Email address: soumyana@ohsu.edu; Telephone: +1-503-494-6878

**A**


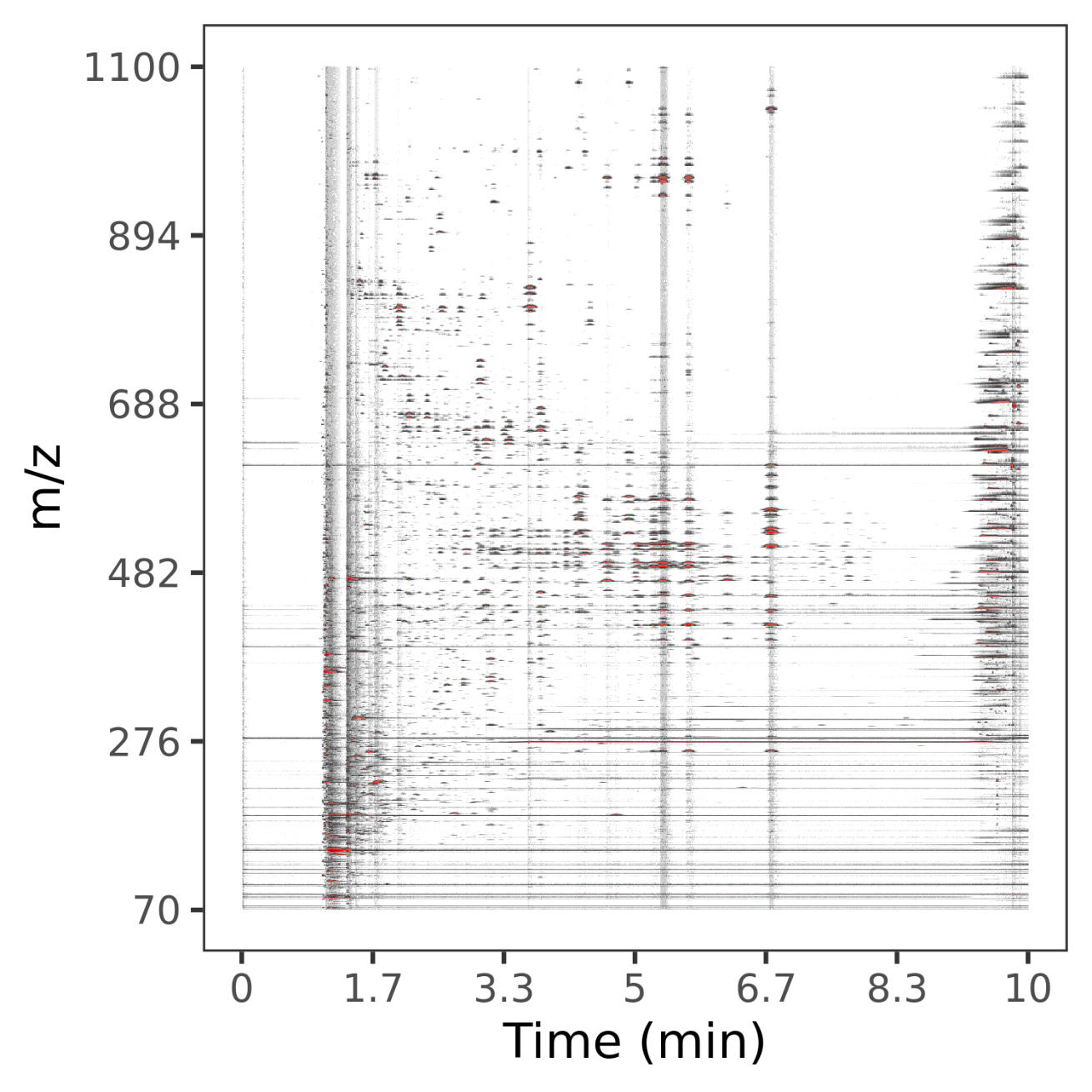


**B**


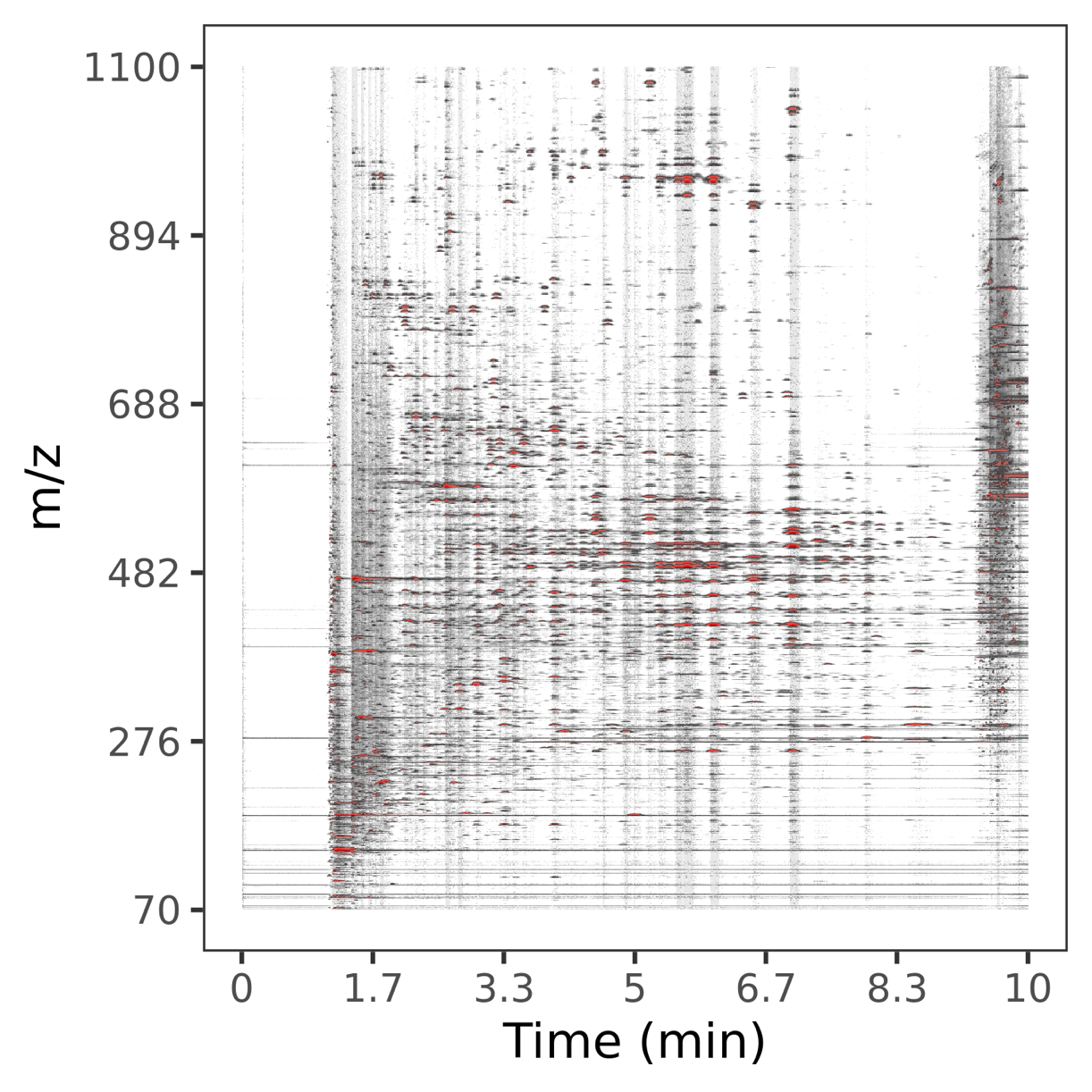


**Figure S1: 2D ion maps for WSAq-9 (A) and WSE-2 (B).**

Data represents LC-HRMS/MS analysis of the extracts (Section 3.4.1) except instrumental analysis was performed on a Sciex 5600 TripleTOF coupled to Shimadzu Nexera UHPLC. Peak intensity is plotted as a heat map, from lowest signal to highest signal, white through grey and finally to red. Plots are on the same scale and dilution (0.25 mg/mL in 70% methanol 30% water and 0.1% formic acid.


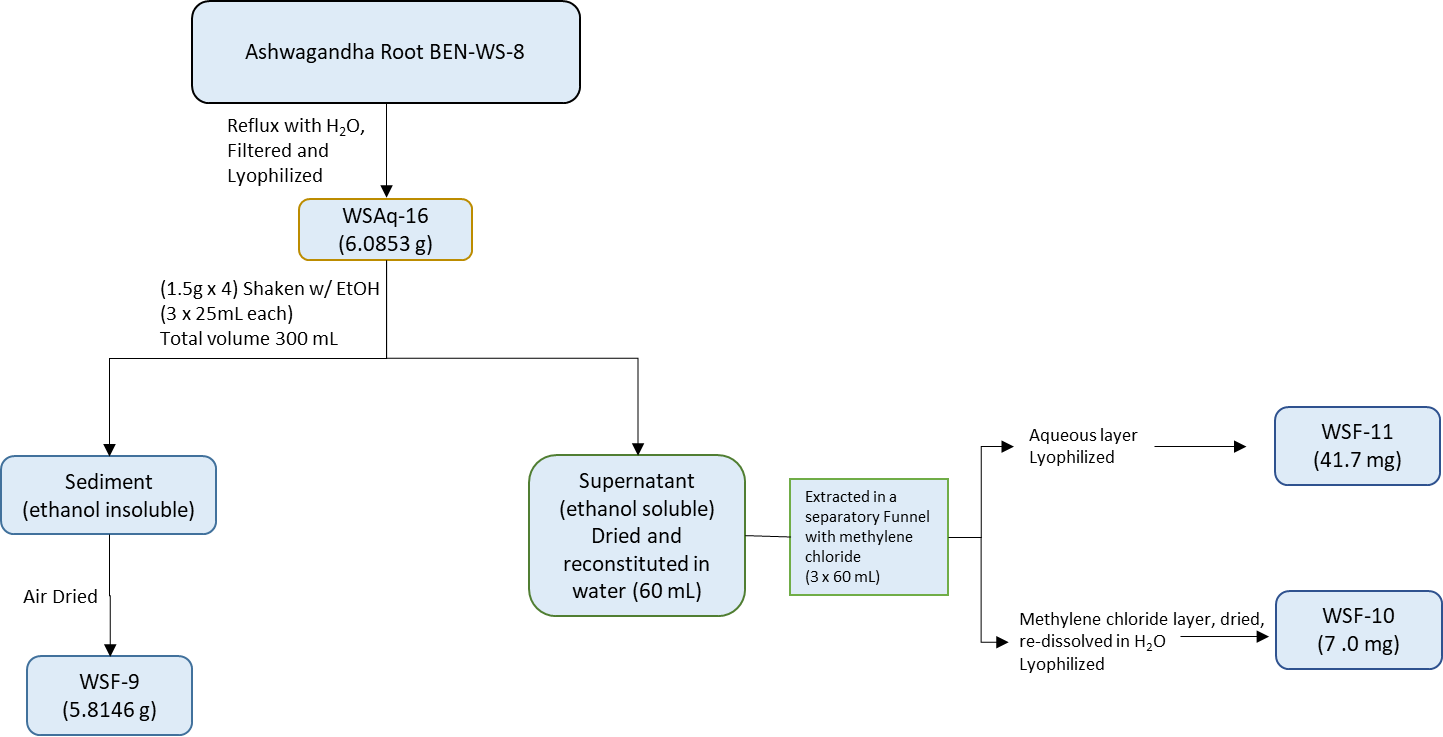


**Figure S2**: **Fractionation scheme for WSAq-16, an aqueous extract of *Withania somnifera* (ashwagandha) root, sample BEN-WS-8.** Three fractions were generated from WSAq-16: WSF-9 (ethanol insoluble fraction), WSF-10 (non-polar fraction) and WSF-11 (polar fraction).


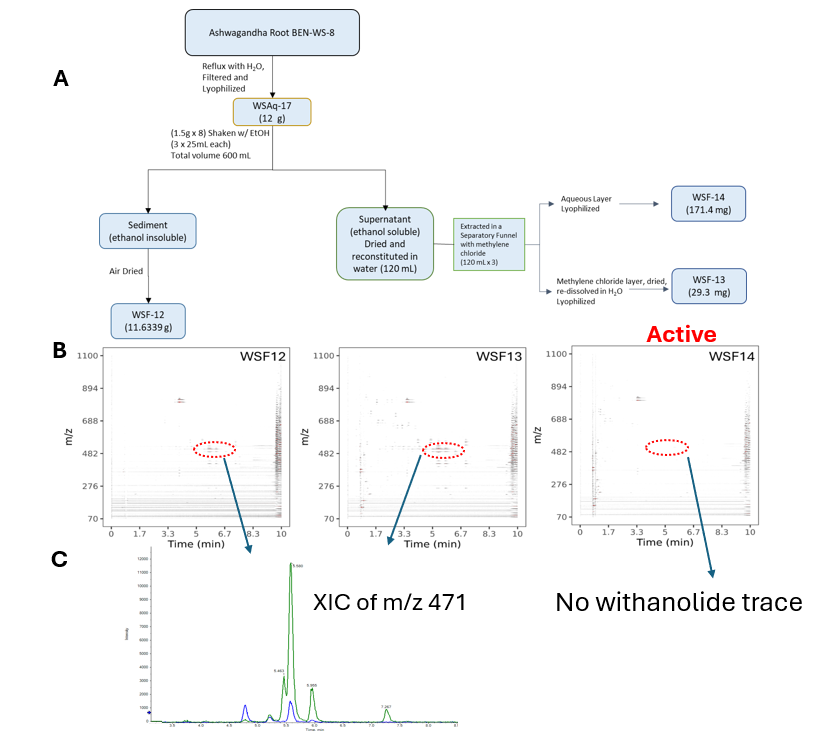


**
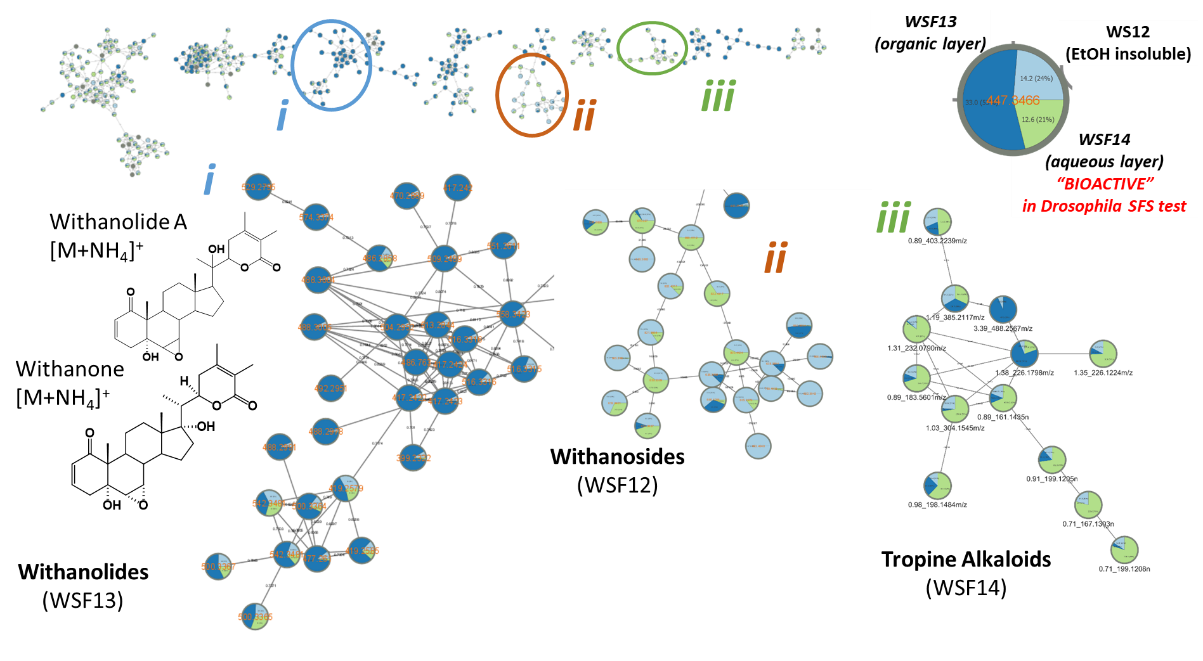
**

**D**

**E**

**
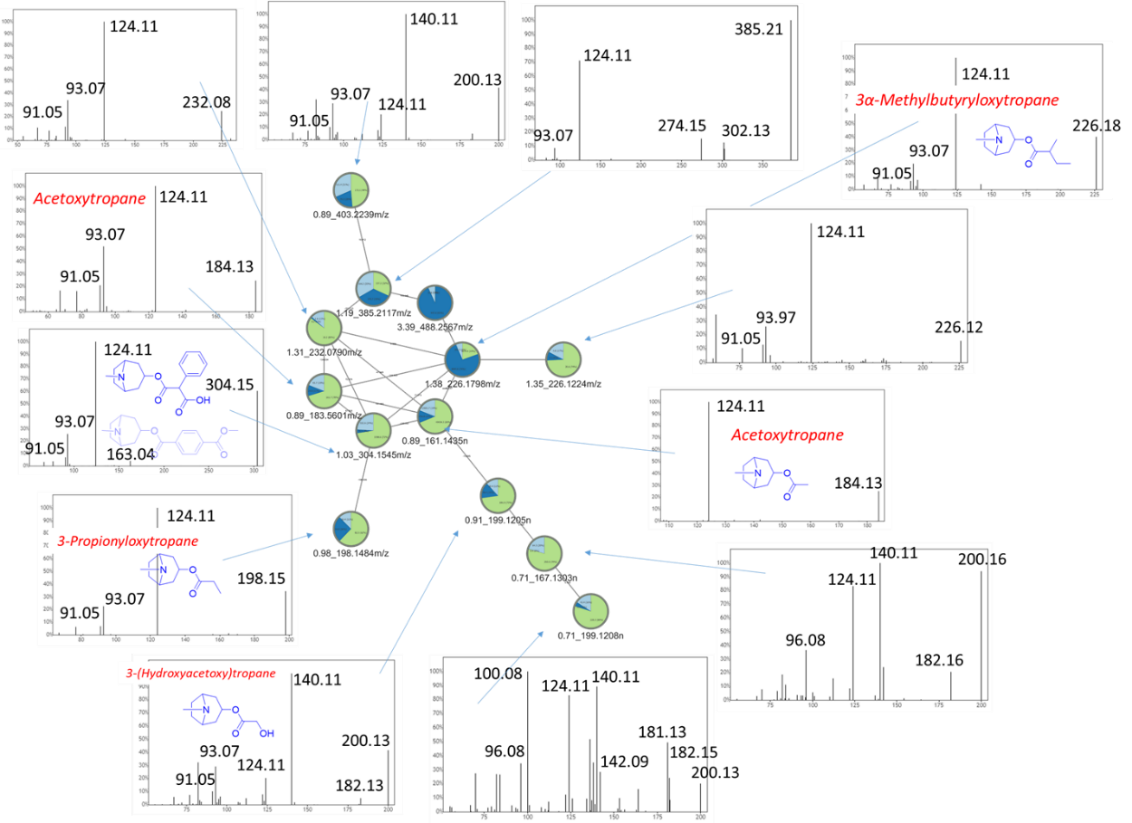
**

**Figure S3: Fractionation of WSAq-17, an aqueous extract of *Withania somnifera* (ashwagandha) root, sample BEN-WS-8 and associated phytochemical analysis of fraction WSF-14 by LC-HRMS/MS (A**) Fractionation scheme of WSAq-17 to WSF-12 (ethanol insoluble fraction), WSF-13 (non-polar fraction) and WSF-14 (polar fraction). (**B**) LC-HRMS/MS ion maps show the absence of withanolides in the bioactive fraction WSF-14. WSF-12 and WSF-13 both contain withanolides. (**C**) The extracted ion chromatogram of WSF-12 (blue line) and WSF-13 (green line) showing the presence of withanolide isomers that have the mass-to-charge ratio (m/z) 471. **(D)** The tandem mass spectrometry (MS/MS) data was used to generate a fragment ion similarity based molecular network on the Global Natural Product Social molecular networking platform. Each node represents precursor ion information, and edges can be denoted as the mass difference between the precursor and similarity measure. GNPS assumes that under similar experimental and instrumental condition two similar structure generate identical fragment ion if not differing by two or more chemical moiety. The data was filtered by removing all MS/MS fragment ions within +/- 17 Da of the precursor m/z. The Top 6 fragment ions in the +/-50 Da window through the spectrum. The precursor ion mass and MS/MS fragment ion mass tolerance was set to 0.05 Da. GNPS molecular networking based on MS/MS spectral similarity suggest presence of tropane alkaloids in aqueous WS root extract (WSAq17) and fraction WSF14, the fraction that was active in the Drosophila SFS test. Withanolides and withanosides were found predominantly in fractions WSF-13 and WSF-12 respectively. **(E)** The molecular network was generated using cosine similarity of fragment ion at 0.7 threshold with more than 6 fragment ion matching. Two nodes remaining edge joined if and only if each of the nodes appeared in each other’s top 10 most similar nodes. The maximum size of the nodes in the network is set to 100. The Cytoscape preview data was downloaded from the GNPS platform and pie chart were created by choosing the fraction number 12, 13 and 14. Tropane alkaloids show distinct fragment ion peaks at m/z 91, 93 and 124. Nodes are annotated with the neutral mass of the precursor ion or with the observed m/z values. .


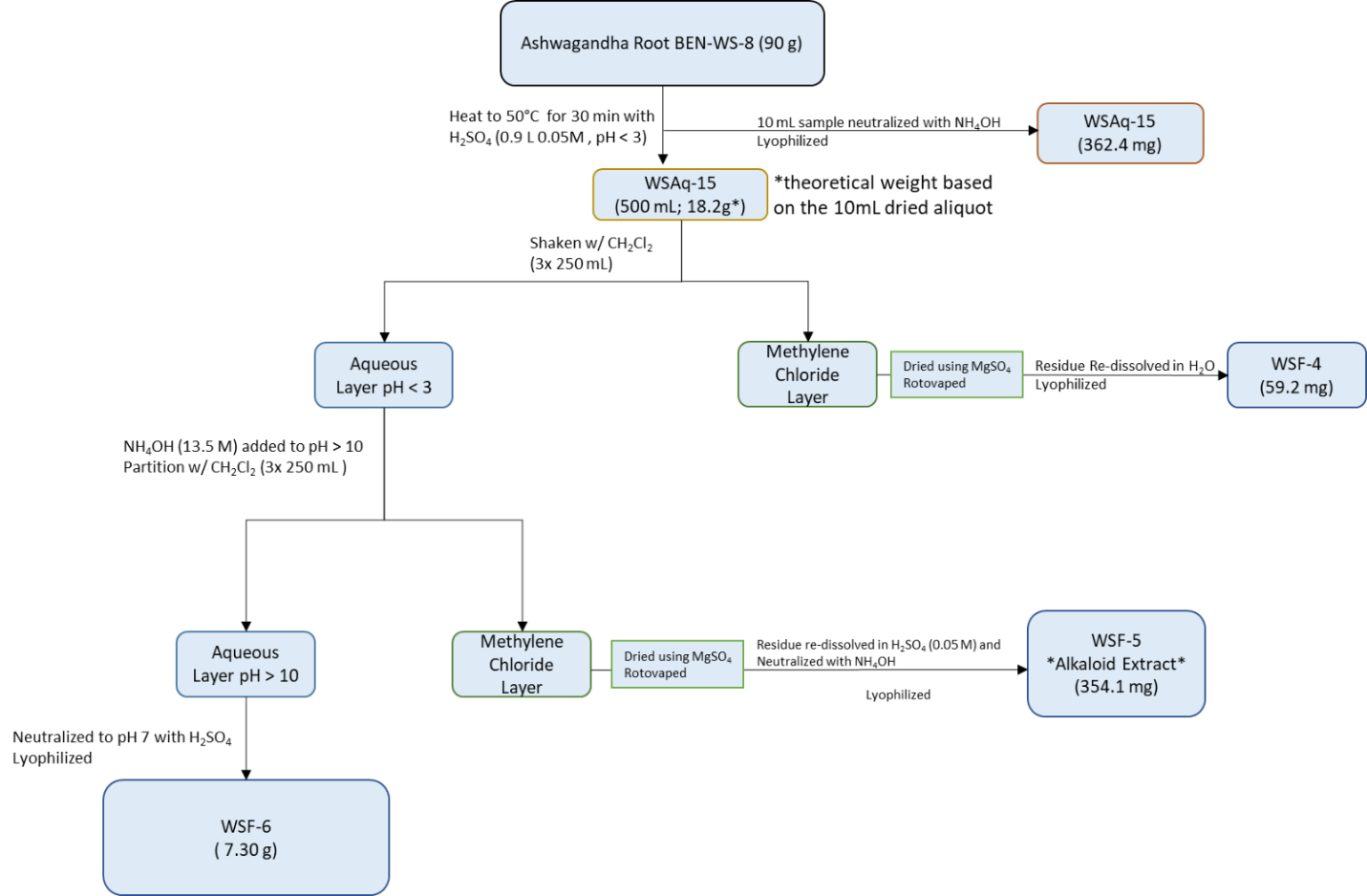


**Figure S4:** Fractionation scheme for preparation of an alkaloidal extract (WSF-5) from ashwagandha root sample BEN-WS-8.


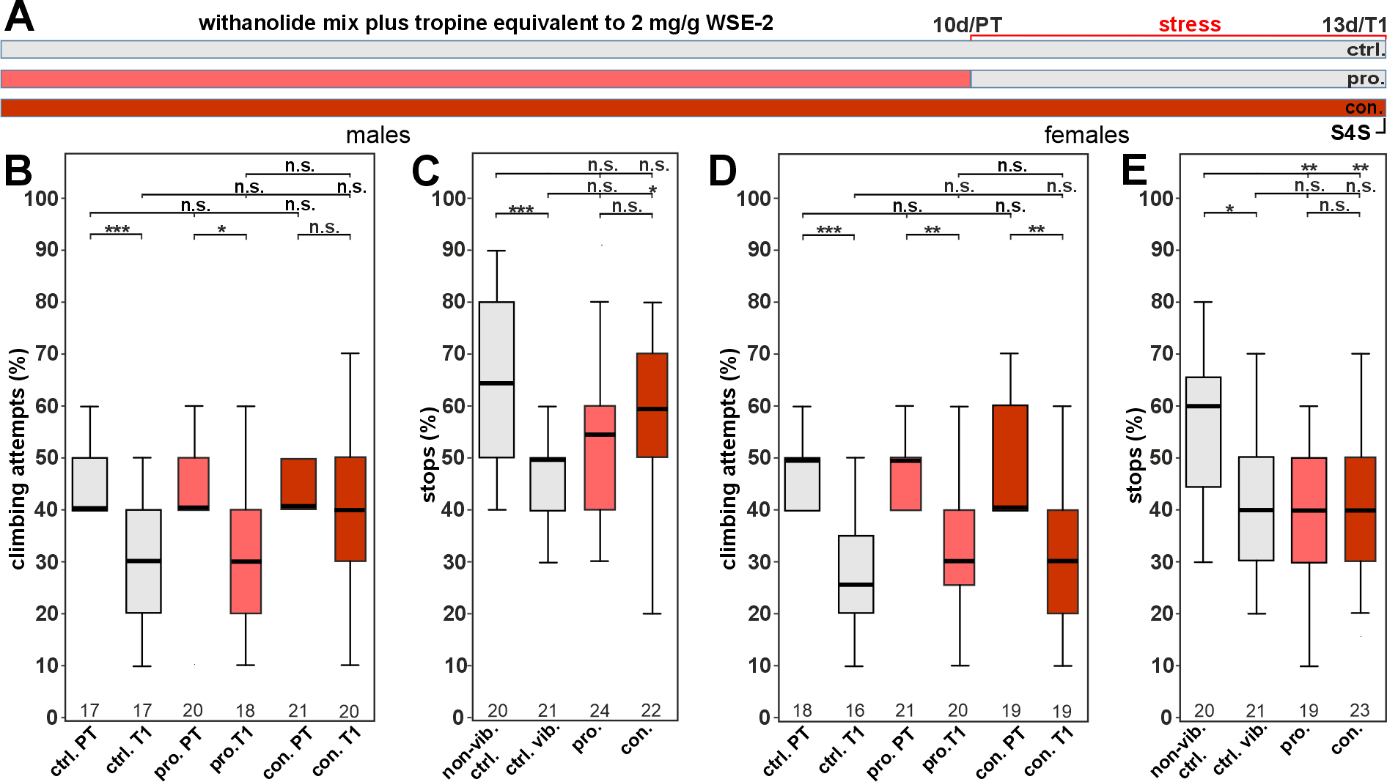


**Figure S5: A withanolide mix with added tropine provides resilience to male flies when administered continuously before and after stress.**

(A) Treatment outline schematic: the time of treatment with the compound mix equivalent to 2 mg/g WSE-2 is shown in red, time of food administration without the mix is shown in gray. (B) Percentage of gap-climbing attempts of males before (PT) and after (T1) stress exposure. Continuously treated males (dark red) were not significantly affected by the stress because they performed equally well after the stress was applied (T1) as before (PT). But they were not significantly different from stressed untreated males (ctrl. T1, gray).(C) Percentage of stops of males at a sweet-tasting stripe. Stressed males receiving control treatment perform significantly worse than the unstressed control (non-vib.). Stressed males receiving prophylactic or continuous treatment with the mix are not different from the unstressed control, and stressed males receiving continuous treatment show improved S4S compared to stressed control flies. Treatment of females did not result in an improvement in gap-climbing (D) or in the S4S test (E). A pairwise Wilcoxon test with Bonferroni–Holm correction for multiple comparisons was used in each panel. The number of analyzed flies is given below the boxes. The horizontal bars in the box plots represent the medians, boxes the 25% and 75% quartiles, and whiskers the data points within ± 1.5 times the interquartile range (IQR). Abbreviations: S4S =stop for sweets.

**Table S1: Comparison of fraction yields and concentrations tested in the bioassays.**

| Test material | Source | Relative weight | Relative % | Test concentration |
| --- | --- | --- | --- | --- |
| Primary Neuron experiment: WSAq16 fractionation Figure S2; Bioassay Figure 1 | | | | |
| WSAq16 | Water extract of *W.somnifera* root | 6.08 g was fractionated | 100% | 100 µg/mL |
| WSF-9 | WSAq16 | 5.8146 g | 95.5% | 95.5 µg/mL |
| WSF-10 | WSAq16 | 0.0070 g | 0.12% | 0.12 µg/mL |
| WSF-11 | WSAq16 | 0.0417 g | 0.69% | 0.69 µg/mL |
|  |  |  |  |  |
| Drosophila experiment: WSAq17 fractionation Figure S3A; Bioassay Figure 3 | | | | |
| WSAq17 | Water extract of *W.somnifera* root | 12.0g was fractionated | 100% | 500 µg/g |
| WSF-12 | WSAq17 | 11.6339 g | 96.9% | 500 µg/g |
| WSF-13 | WSAq17 | 0.0293 g | 0.24% | 1 µg/g |
| WSF-14 | WSAq17 | 0.1714 g | 1.42% | 7 µg/g |
|  |  |  |  |  |
| Drosophila experiment: WSAq15 fractionation Figure S4; Bioassay Figure 6 | | | | |
| WSAq15 | Acid extract of *W.somnifera* root | 18.12 g was fractionated | 100% | not tested |
| WSF-5 | WSAq15 | 0.3541 g | 1.95% | 10 µg/g |
|  |  |  |  |  |

For fractions WSF9- WSF14, the concentrations tested were relative to bioactive concentrations of the parent extracts (WSAq 16 or WSAq17). The alkaloidal extract WSF-5 comprised about 2% w/w of its parent aqueous acid extract WSAq-15. The test concentration of WSF-5 (1 µg/g ) was matched to 2% of the test concentration of aqueous extract WSAq-17 (500 µg/g); WSAq-15 was not tested.

**Table S2: Details of sample size and statistical comparisons for the primary neuron experiment shown in Figure 1**

| Figure # | Groups | Mean value of AUC | SEM | # of neurons assessed | P value and comparison |
| --- | --- | --- | --- | --- | --- |
| 1A | Control | 136.84 | 3.47 | 483 | - |
|  | WSE-2 50 | 144.28 | 3.33 | 628 | ns |
|  | WSE-2 100 | 124.81 | 4.15 | 341 | p=0.024 vs control |
|  | WSAq-9 50 | 139.31 | 3.65 | 318 | ns |
|  | WSAq-9 100 | 164.74 | 3.67 | 340 | p<0.0001 vs control |
|  | WSAq-9 Mix | 143.96 | 2.05 | 537 | ns |
| 1B | Control | 90.07 | 2.21 | 496 | - |
|  | WSAq-16 | 116.48 | 5.03 | 374 | p<0.001 vs control |
|  | WS-F9 | 89.28 | 2.18 | 491 | p=0.003 vs F11; p=<0.0001 vs WSAq16 |
|  | WS-F10 | 89.94 | 2.75 | 317 | p=0.021 vs F11; p<0.0001 vs WSAq16 |
|  | WS-F11 | 101.30 | 2.81 | 320 | p=0.027 vs control; p<0.0001 vs WSAq16 |

**Table S3: Details of sample size (number of flies) and statistical comparisons for the *Drosophila* experiments shown in Figures 2, 3, 6, 8 and S5**

| **Table S3A. Statistics concerning Figure 2.** | | | | | |
| --- | --- | --- | --- | --- | --- |
| **B.** | | | | | |
| **Normal distribution test (Shapiro-Wilk test)** | | | **Statistical comparison of data sets** | | |
| **Group** | **sample size** | **p-value** | **Comparison** | **Stat. test** | **p-value** |
| Gap-climbing ctrl. PT | 22 | 0.0115 | ctrl. PT vs. ctrl. T1 | Wilcox. | 6.13-e05 |
| Gap-climbing ctrl. T1 | 22 | 0.0026 | pro. PT vs. pro. T1 | Wilcox. | 1.40e-04 |
| Gap-climbing pro. PT | 23 | 0.0057 | con. PT vs. con. T1 | Wilcox. | 1.45e-04 |
| Gap-climbing pro. T1 | 21 | 0.0461 | ctrl. PT vs. pro. PT | Wilcox. | 0.731 |
| Gap-climbing con. PT | 21 | 4.92e-05 | ctrl. PT vs. con. PT | Wilcox. | 0.0301 |
| Gap-climbing con. T1 | 20 | 0.162 | ctrl. T1 vs. pro. T1 | Wilcox. | 0.523 |
| **Median rank comparison (Kruskal-Wallis test)** | | | ctrl. T1 vs. con. T1 | Wilcox. | 0.602 |
| All data: p = 3.03e-12 | | | pro. T1 vs. con. T1 | Wilcox. | 0.884 |
| **C.** | | | | | |
| **Normal distribution test (Shapiro-Wilk test)** | | | **Statistical comparison of data sets** | | |
| **Group** | **sample size** | **p-value** | **Comparison** | **Stat. test** | **p-value** |
| non.-vib. | 21 | 0.214 | non.-vib. ctrl. vs. vib. ctrl. | Wilcox. | 1.45e-07 |
| vib. ctrl. | 24 | 0.0455 | non.-vib. ctrl. vs. pro. | Wilcox. | 6.95e-09 |
| pro. | 28 | 9.00e-04 | non.-vib. vs. con. | Wilcox. | 4.29e-06 |
| con. | 23 | 0.0138 | vib. ctrl. vs. pro. | Wilcox. | 0.637 |
| **Median rank comparison (Kruskal-Wallis test)** | | | vib. ctrl. vs. con. | Wilcox. | 0.454 |
| All data: p = 9.03e-07 | | | pro. vs. con. | Wilcox. | 0.215 |
| **D.** | | | | | |
| **Normal distribution test (Shapiro-Wilk test)** | | | **Statistical comparison of data sets** | | |
| **Group** | **sample size** | **p-value** | **Comparison** | **Stat. test** | **p-value** |
| Gap-climbing ctrl. PT | 19 | 0.0019 | ctrl. PT vs. ctrl. T1 | Wilcox. | 1.68e-04 |
| Gap-climbing ctrl. T1 | 17 | 0.161 | pro. PT vs. pro. T1 | Wilcox. | 5.03e-04 |
| Gap-climbing pro. PT | 22 | 4.00e-04 | con. PT vs. con. T1 | Wilcox. | 9.34e-04 |
| Gap-climbing pro. T1 | 21 | 0.0078 | ctrl. PT vs. pro. PT | Wilcox. | 0.763 |
| Gap-climbing con. PT | 20 | 5.00e-04 | ctrl. PT vs. con. PT | Wilcox. | 0.574 |
| Gap-climbing con. T1 | 20 | 0.154 | ctrl. T1 vs. pro. T1 | Wilcox. | 0.266 |
| **Median rank comparison (Kruskal-Wallis test)** | | | ctrl. T1 vs. con. T1 | t-test | 0.865 |
| All data: p = 3.19e-10 | | | pro. T1 vs. con. T1 | Wilcox. | 0.516 |
| **E.** | | | | | |
| **Normal distribution test (Shapiro-Wilk test)** | | | **Statistical comparison of data sets** | | |
| **Group** | **sample size** | **p-value** | **Comparison** | **Stat. test** | **p-value** |
| non.-vib. ctrl. | 21 | 0.311 | non.-vib. ctrl. vs. vib. | t-test | 4.35e-04 |
| vib. ctrl. | 22 | 0.0553 | non.-vib. ctrl. vs. pro. | t-test | 3.50e-05 |
| pro. | 20 | 0.302 | non.-vib. ctrl. vs. con. | t-test | 0.00109 |
| con. | 24 | 0.103 | vib. ctrl. vs. pro. | t-test | 0.435 |
| **Median rank comparison (Kruskal-Wallis test)** | | | vib. ctrl. vs. con. | t-test | 0.723 |
| All data: p = 1.86e-04 | | | pro. vs. con. | t-test | 0.255 |

| **Table S3B. Statistics concerning Figure 3.** | | | | | | |
| --- | --- | --- | --- | --- | --- | --- |
| **A. (Gap climbing)** | | | | | | |
| **Normal distribution test (Shapiro-Wilk test)** | | | **Statistical comparison of data sets** | | | |
| **Group** | **sample size** | **p-value** | **Comparison** | **Stat. test** | **p-value** | |
| Gap-climbing ctrl. PT | 20 | 2.73e-04 | ctrl. PT vs. ctrl. T1 | Wilcox. | 5.81e-05 | |
| Gap-climbing ctrl. T1 | 20 | 0.214 | pro. PT vs. pro. T1 | Wilcox. | 0.072 | |
| Gap-climbing pro. PT | 22 | 4.17e-04 | con. PT vs. con. T1 | Wilcox. | 0.169 | |
| Gap-climbing pro. T1 | 21 | 0.00369 | ctrl. PT vs. pro. PT | Wilcox. | 0.941 | |
| Gap-climbing con. PT | 20 | 2.46e-04 | ctrl. PT vs. con. PT | Wilcox. | 0.496 | |
| Gap-climbing con. T1 | 20 | 0.505 | ctrl. T1 vs. pro. T1 | Wilcox. | 0.00219 | |
| **Median rank comparison (Kruskal-Wallis test)** | | | ctrl. T1 vs. con. T1 | t-test | 3.12e-05 | |
| All data: p = 6.39e-07 | | | pro. T1 vs. con. T1 | Wilcox. | 0.248 | |
| **A. (SFS)** | | | | | | |
| **Normal distribution test (Shapiro-Wilk test)** | | | **Statistical comparison of data sets** | | | |
| **Group** | **sample size** | **p-value** | **Comparison** | **Stat. test** | **p-value** | |
| vib. ctrl. | 23 | 0.0433 | vib. ctrl. vs. pro. | Wilcox. | 8.48e-05 | |
| pro. | 22 | 0.427 | vib. ctrl. vs. con. | Wilcox. | 3.23e-06 | |
| con. | 23 | 0.178 | pro. vs. con. | t-test | 0.256 | |
| **Median rank comparison (Kruskal-Wallis test)** | | |  | | | |
| All data: p = 3.44e-07 | | |  |  |  |  |
| **B. (Gap climbing)** | | | | | | |
| **Normal distribution test (Shapiro-Wilk test)** | | | **Statistical comparison of data sets** | | | |
| **Group** | **sample size** | **p-value** | **Comparison** | **Stat. test** | **p-value** | |
| Gap-climbing ctrl. PT | 21 | 8.56e-06 | ctrl. PT vs. ctrl. T1 | Wilcox. | 4.50e-05 | |
| Gap-climbing ctrl. T1 | 20 | 0.351 | pro. PT vs. pro. T1 | Wilcox. | 0.00132 | |
| Gap-climbing pro. PT | 21 | 3.79e-05 | con. PT vs. con. T1 | Wilcox. | 0.0102 | |
| Gap-climbing pro. T1 | 20 | 0.0974 | ctrl. PT vs. pro. PT | Wilcox. | 0.490 | |
| Gap-climbing con. PT | 20 | 1.25e-04 | ctrl. PT vs. con. PT | Wilcox. | 0.366 | |
| Gap-climbing con. T1 | 19 | 0.0463 | ctrl. T1 vs. pro. T1 | t-test | 0.321 | |
| **Median rank comparison (Kruskal-Wallis test)** | | | ctrl. T1 vs. con. T1 | Wilcox. | 0.0641 | |
| All data: p = 6.97e-10 | | | pro. T1 vs. con. T1 | Wilcox. | 0.366 | |
| **B. (SFS)** | | | | | | |
| **Normal distribution test (Shapiro-Wilk test)** | | | **Statistical comparison of data sets** | | | |
| **Group** | **sample size** | **p-value** | **Comparison** | **Stat. test** | **p-value** | |
| vib. ctrl. | 22 | 0.111 | vib. ctrl. vs. pro. | t-test | 0.420 | |
| pro. | 21 | 0.248 | vib. ctrl. vs. con. | t-test | 0.0136 | |
| con. | 22 | 0.0545 | pro. vs. con. | t-test | 0.0678 | |
| **Median rank comparison (Kruskal-Wallis test)** | | |  | | | |
| All data: p = 0.00878 | | |  |  |  |  |
| **C. (Gap climbing)** | | | | | | |
| **Normal distribution test (Shapiro-Wilk test)** | | | **Statistical comparison of data sets** | | | |
| **Group** | **sample size** | **p-value** | **Comparison** | **Stat. test** | **p-value** | |
| Gap-climbing ctrl. PT | 16 | 8.48e-04 | ctrl. PT vs. ctrl. T1 | Wilcox. | 2.91e-04 | |
| Gap-climbing ctrl. T1 | 16 | 0.00575 | pro. PT vs. pro. T1 | Wilcox. | 0.00972 | |
| Gap-climbing pro. PT | 21 | 1.049e-04 | con. PT vs. con. T1 | Wilcox. | 2.45e-05 | |
| Gap-climbing pro. T1 | 21 | 0.479 | ctrl. PT vs. pro. PT | Wilcox. | 0.999 | |
| Gap-climbing con. PT | 20 | 6.64e-04 | ctrl. PT vs. con. PT | Wilcox. | 0.833 | |
| Gap-climbing con. T1 | 20 | 0.0886 | ctrl. T1 vs. pro. T1 | Wilcox. | 0.379 | |
| **Median rank comparison (Kruskal-Wallis test)** | | | ctrl. T1 vs. con. T1 | Wilcox. | 0.840 | |
| All data: p = 1.74e-10 | | | pro. T1 vs. con. T1 | t-test | 0.402 | |
| **C. (SFS)** | | | | | | |
| **Normal distribution test (Shapiro-Wilk test)** | | | **Statistical comparison of data sets** | | | |
| **Group** | **sample size** | **p-value** | **Comparison** | **Stat. test** | **p-value** | |
| vib. ctrl. | 20 | 0.656 | vib. ctrl. vs. pro. | t-test | 0.545 | |
| pro. | 22 | 0.535 | vib. ctrl. vs. con. | t-test | 0.0929 | |
| con. | 21 | 0.248 | pro. vs. con. | t-test | 0.758 | |
| **Median rank comparison (Kruskal-Wallis test)** | | |  | | | |
| All data: p = 0.119 | | |  |  |  |  |
| **D. (Gap climbing)** | | | | | | |
| **Normal distribution test (Shapiro-Wilk test)** | | | **Statistical comparison of data sets** | | | |
| **Group** | **sample size** | **p-value** | **Comparison** | **Stat. test** | **p-value** | |
| Gap-climbing ctrl. PT | 21 | 1.05e-04 | ctrl. PT vs. ctrl. T1 | Wilcox. | 1.03e-04 | |
| Gap-climbing ctrl. T1 | 20 | 0.00959 | pro. PT vs. pro. T1 | Wilcox. | 0.0475 | |
| Gap-climbing pro. PT | 21 | 0.00221 | con. PT vs. con. T1 | Wilcox. | 0.0791 | |
| Gap-climbing pro. T1 | 21 | 0.0313 | ctrl. PT vs. pro. PT | Wilcox. | 0.540 | |
| Gap-climbing con. PT | 21 | 1.89e-05 | ctrl. PT vs. con. PT | Wilcox. | 0.775 | |
| Gap-climbing con. T1 | 20 | 0.108 | ctrl. T1 vs. pro. T1 | Wilcox. | 0.911 | |
| **Median rank comparison (Kruskal-Wallis test)** | | | ctrl. T1 vs. con. T1 | Wilcox. | 0.0777 | |
| All data: p = 5.72e-07 | | | pro. T1 vs. con. T1 | Wilcox. | 0.860 | |
| **D. (SFS)** | | | | | | |
| **Normal distribution test (Shapiro-Wilk test)** | | | **Statistical comparison of data sets** | | | |
| **Group** | **sample size** | **p-value** | **Comparison** | **Stat. test** | **p-value** | |
| vib. ctrl. | 22 | 0.0263 | vib. ctrl. vs. pro. | Wilcox. | 2.86e-05 | |
| pro. | 23 | 0.0230 | vib. ctrl. vs. con. | Wilcox. | 2.58e-04 | |
| con. | 22 | 0.0502 | pro. vs. con. | Wilcox. | 0.999 | |
| **Median rank comparison (Kruskal-Wallis test)** | | |  | | | |
| All data: p = 1.56e-05 | | |  |  |  |  |

| **Table S3C. Statistics concerning Figure 6.** | | | | | |
| --- | --- | --- | --- | --- | --- |
| **A.** | | | | | |
| **Normal distribution test (Shapiro-Wilk test)** | | | **Statistical comparison of data sets** | | |
| **Group** | **sample size** | **p-value** | **Comparison** | **Stat. test** | **p-value** |
| Gap-climbing ctrl. PT | 20 | 8.08e-07 | ctrl. PT vs. ctrl. T1 | Wilcox. | 3.99e-06 |
| Gap-climbing ctrl. T1 | 20 | 0.0027 | pro. PT vs. pro. T1 | Wilcox. | 0.260 |
| Gap-climbing pro. PT | 22 | 0.0019 | con. PT vs. con. T1 | Wilcox. | 0.708 |
| Gap-climbing pro. T1 | 22 | 0.0069 | ctrl. PT vs. pro. PT | Wilcox. | 0.276 |
| Gap-climbing con. PT | 21 | 1.92e-06 | ctrl. PT vs. con. PT | Wilcox. | 0.574 |
| Gap-climbing con. T1 | 20 | 0.262 | ctrl. T1 vs. pro. T1 | Wilcox. | 1.49e-05 |
| **Median rank comparison (Kruskal-Wallis test)** | | | ctrl. T1 vs. con. T1 | Wilcox. | 0.00140 |
| All data: p = 4.15e-09 | | | pro. T1 vs. con. T1 | Wilcox. | 0.319 |
| **B.** | | | | | |
| **Normal distribution test (Shapiro-Wilk test)** | | | **Statistical comparison of data sets** | | |
| **Group** | **sample size** | **p-value** | **Comparison** | **Stat. test** | **p-value** |
| non.-vib. | 19 | 0.155 | non.-vib. ctrl. vs. vib. ctrl. | t-test | 1.34e-04 |
| vib. ctrl. | 21 | 0.346 | non.-vib. ctrl. vs. pro. | t-test | 0.977 |
| pro. | 20 | 0.173 | non.-vib. vs. con. | t-test | 0.783 |
| con. | 19 | 0.0893 | vib. ctrl. vs. pro. | t-test | 1.16e-04 |
| **Median rank comparison (Kruskal-Wallis test)** | | | vib. ctrl. vs. con. | t-test | 1.06e-04 |
| All data: p = 2.27e-06 | | | pro. vs. con. | t-test | 0.783 |

| **Table S3D. Statistics concerning Figure 8.** | | | | | |
| --- | --- | --- | --- | --- | --- |
| **A.** | | | | | |
| **Normal distribution test (Shapiro-Wilk test)** | | | **Statistical comparison of data sets** | | |
| **Group** | **sample size** | **p-value** | **Comparison** | **Stat. test** | **p-value** |
| Gap-climbing ctrl. PT | 24 | 1.40e-04 | ctrl. PT vs. ctrl. T1 | Wilcox. | 1.96e-07 |
| Gap-climbing ctrl. T1 | 24 | 0.00808 | pro. PT vs. pro. T1 | Wilcox. | 0.503 |
| Gap-climbing pro. PT | 20 | 1.86e-06 | con. PT vs. con. T1 | Wilcox. | 0.0647 |
| Gap-climbing pro. T1 | 19 | 0.428 | ctrl. PT vs. pro. PT | Wilcox. | 0.479 |
| Gap-climbing con. PT | 20 | 5.24e-05 | ctrl. PT vs. con. PT | Wilcox. | 0.725 |
| Gap-climbing con. T1 | 20 | 0.0471 | ctrl. T1 vs. pro. T1 | Wilcox. | 0.0155 |
| **Median rank comparison (Kruskal-Wallis test)** | | | ctrl. T1 vs. con. T1 | Wilcox. | 0.00398 |
| All data: p = 6.17e-10 | | | pro. T1 vs. con. T1 | Wilcox. | 0.829 |
| **B.** | | | | | |
| **Normal distribution test (Shapiro-Wilk test)** | | | **Statistical comparison of data sets** | | |
| **Group** | **sample size** | **p-value** | **Comparison** | **Stat. test** | **p-value** |
| non.-vib. | 24 | 0.0567 | non.-vib. ctrl. vs. vib. ctrl. | t-test | 0.00251 |
| vib. ctrl. | 22 | 0.144 | non.-vib. ctrl. vs. pro. | t-test | 0.548 |
| pro. | 26 | 0.686 | non.-vib. vs. con. | t-test | 0.894 |
| con. | 22 | 0.0589 | vib. ctrl. vs. pro. | t-test | 0.0386 |
| **Median rank comparison (Kruskal-Wallis test)** | | | vib. ctrl. vs. con. | t-test | 0.0184 |
| All data: p = 0.00282 | | | pro. vs. con. | t-test | 0.548 |

| **Table S3E. Statistics concerning Figure S5.** | | | | | |
| --- | --- | --- | --- | --- | --- |
| **B.** | | | | | |
| **Normal distribution test (Shapiro-Wilk test)** | | | **Statistical comparison of data sets** | | |
| **Group** | **sample size** | **p-value** | **Comparison** | **Stat. test** | **p-value** |
| Gap-climbing ctrl. PT | 17 | 2.038e-04 | ctrl. PT vs. ctrl. T1 | Wilcox. | 9.17e-04 |
| Gap-climbing ctrl. T1 | 17 | 0.142 | pro. PT vs. pro. T1 | Wilcox. | 0.0128 |
| Gap-climbing pro. PT | 20 | 8.25e-05 | con. PT vs. con. T1 | Wilcox. | 0.203 |
| Gap-climbing pro. T1 | 18 | 0.0221 | ctrl. PT vs. pro. PT | Wilcox. | 0.876 |
| Gap-climbing con. PT | 21 | 3.21e-06 | ctrl. PT vs. con. PT | Wilcox. | 0.657 |
| Gap-climbing con. T1 | 20 | 0.500 | ctrl. T1 vs. pro. T1 | Wilcox. | 0.708 |
| **Median rank comparison (Kruskal-Wallis test)** | | | ctrl. T1 vs. con. T1 | t-test | 0.0102 |
| All data: p = 2.64e-06 | | | pro. T1 vs. con. T1 | Wilcox. | 0.0529 |
| **C.** | | | | | |
| **Normal distribution test (Shapiro-Wilk test)** | | | **Statistical comparison of data sets** | | |
| **Group** | **sample size** | **p-value** | **Comparison** | **Stat. test** | **p-value** |
| non.-vib. | 20 | 0.112 | non.-vib. ctrl. vs. vib. ctrl. | t-test | 3.76e-04 |
| vib. ctrl. | 21 | 0.242 | non.-vib. ctrl. vs. pro. | t-test | 0.0902 |
| pro. | 24 | 0.113 | non.-vib. vs. con. | t-test | 0.353 |
| con. | 22 | 0.230 | vib. ctrl. vs. pro. | t-test | 0.125 |
| **Median rank comparison (Kruskal-Wallis test)** | | | vib. ctrl. vs. con. | t-test | 0.0466 |
| All data: p = 0.00295 | | | pro. vs. con. | t-test | 0.579 |
| **D.** | | | | | |
| **Normal distribution test (Shapiro-Wilk test)** | | | **Statistical comparison of data sets** | | |
| **Group** | **sample size** | **p-value** | **Comparison** | **Stat. test** | **p-value** |
| Gap-climbing ctrl. PT | 18 | 0.00294 | ctrl. PT vs. ctrl. T1 | Wilcox. | 1.69e-04 |
| Gap-climbing ctrl. T1 | 16 | 0.161 | pro. PT vs. pro. T1 | Wilcox. | 0.00156 |
| Gap-climbing pro. PT | 21 | 1.67e-04 | con. PT vs. con. T1 | Wilcox. | 0.00257 |
| Gap-climbing pro. T1 | 20 | 0.182 | ctrl. PT vs. pro. PT | Wilcox. | 0.564 |
| Gap-climbing con. PT | 19 | 1.68e-04 | ctrl. PT vs. con. PT | Wilcox. | 0.987 |
| Gap-climbing con. T1 | 19 | 0.363 | ctrl. T1 vs. pro. T1 | t-test | 0.156 |
| **Median rank comparison (Kruskal-Wallis test)** | | | ctrl. T1 vs. con. T1 | t-test | 0.299 |
| All data: p = 1.34e-09 | | | pro. T1 vs. con. T1 | t-test | 0.685 |
| **E.** | | | | | |
| **Normal distribution test (Shapiro-Wilk test)** | | | **Statistical comparison of data sets** | | |
| **Group** | **sample size** | **p-value** | **Comparison** | **Stat. test** | **p-value** |
| non.-vib. ctrl. | 20 | 0.311 | non.-vib. ctrl. vs. vib. | t-test | 0.0212 |
| vib. ctrl. | 21 | 0.164 | non.-vib. ctrl. vs. pro. | t-test | 0.00289 |
| pro. | 21 | 0.158 | non.-vib. ctrl. vs. con. | t-test | 0.00227 |
| con. | 23 | 0.0922 | vib. ctrl. vs. pro. | t-test | 0.366 |
| **Median rank comparison (Kruskal-Wallis test)** | | | vib. ctrl. vs. con. | t-test | 0.561 |
| All data: p = 0.00367 | | | pro. vs. con. | t-test | 0.672 |

**Table S4: Experimental details of mass spectrometry conditions used in LC-MRM-MS of tropane alkaloids** (main text section 3.5.1)

| Compound | Formula | Q1 (m/z) | Q3 (m/z) | CE | CV | Retention time (min) |
| --- | --- | --- | --- | --- | --- | --- |
| Acetyltropine | C_10_H_17_NO_2_ | 184.0 | 124.06 | 18 | 4 | 1.82 |
| Atropine-d_5_ | C_17_H_18_D_5_NO_3_ | 295.4 | 124.06 | 24 | 30 | 6.73 |
| Pseudotropine | C_8_H_15_NO | 142.21 | 67.14 | 24 | 100 | 0.82 |
| Tigloyloxytropane | C_13_H_21_NO_2_ | 224.31 | 124.06 | NA | NA | 7.03 |
| Tropine | C_8_H_15_NO | 142.21 | 67.08 | 24 | 42 | 0.82 |

Q1: Precursor ion Q3: Quantifier ion CV: Cone Voltage CE: Collision Energy


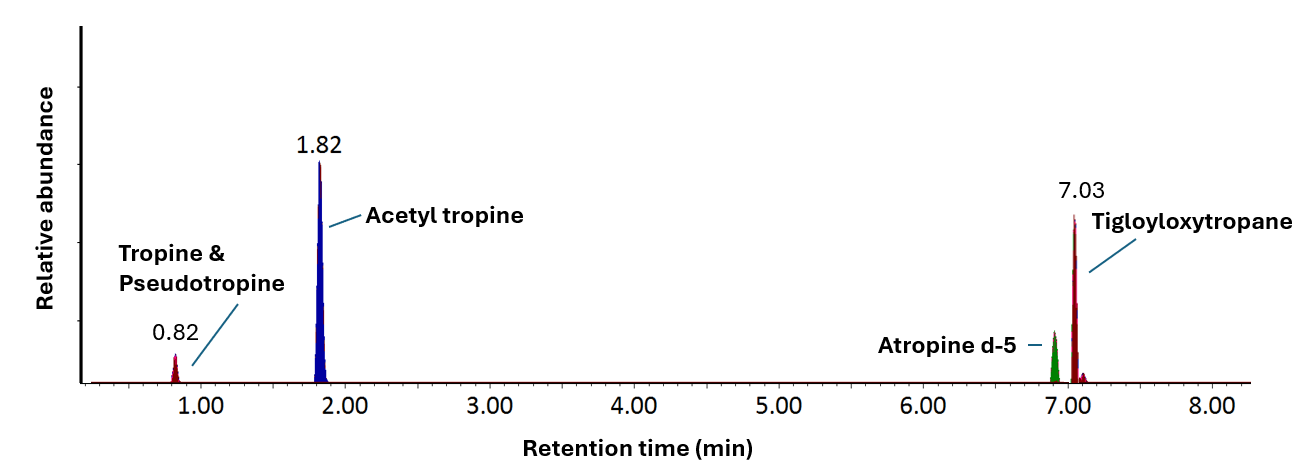


**Figure S6**: Chromatogram showing chromatographic separation of three alkaloids in BEN WS-8 (root) sample using LC-MRM-MS. Atropine-d_5_ was used as an internal standard.

**Table S5: Alkaloids detected in WSF14 using LC-HRMS/MS and their annotation levels**

(to support Figure 5 in the main section)

| Name | Formula | Exact m/z | Measured m/z | Mass defect | Annota-tion level | Fragment ion m/z |
| --- | --- | --- | --- | --- | --- | --- |
| Tropane | C8H15N | 126.2196 | 126.1277 | 0.1277 | 4 |  |
| Norpseudo-tropine | C7H13NO | 128.1924 | 128.107 | 0.107 | 4 |  |
| Tropinone | C8H13NO | 140.2032 | 140.107 | 0.107 | 4 |  |
| Hygrine | C8H15NO | 142.219 | 142.1226 | 0.1226 | 5 |  |
| Tropine | C8H15NO | 142.219 | 142.1226 | 0.1226 | 2 | 96.0609 |
| Pseudotropine | C8H15NO | 142.219 | 142.1226 | 0.1226 | 2 | 96.0609 |
| Calystegine A3 | C7H13NO3 | 160.1912 | 160.0968 | 0.0968 | 5 |  |
| Swainsonine | C8H15NO3 | 174.2178 | 174.1125 | 0.1125 | 5 |  |
| Calystegine B2 | C7H13NO4 | 176.1906 | 176.0917 | 0.0917 | 5 |  |
| Acetoxytropane | C10H17NO2 | 184.2558 | 184.1332 | 0.1332 | 1 | 124.1119 |
| Castanospermine | C8H15NO4 | 190.2173 | 190.1074 | 0.1074 | 5 |  |
| 3-Hydroxyacetoxy)-tropane | C10H17NO3 | 200.2552 | 200.1281 | 0.1281 | 4 |  |
| 3-Propionyloxy-tropane | C11H19NO2 | 198.2824 | 198.1494 | 0.1494 | 5 |  |
| 3- (3`Methoxy-  tropoyloxy)tropane | C10H17NO3 | 200.2552 | 200.1286 | 0.1286 | 5 |  |
| 3α-Methyl-butyryloxytropane | C13H23NO2 | 226.3356 | 226.1807 | 0.1807 | 5 |  |
| 6,7-Dehydro-3-tigloyloxytropane | C13H19NO2 | 222.3039 | 222.1489 | 0.1489 | 5 |  |
| Tigloyloxytropane | C13H21NO2 | 224.3198 | 224.1645 | 0.1645 | 2 | 124.1099 |
| Anaferine | C13H24N2O | 225.3509 | 225.1961 | 0.1961 | 4 |  |
| 3-Hydroxy-6-iso-butyryloxytropane | C12H21NO3 | 228.3084 | 228.1594 | 0.1594 | 5 |  |
| 3-Tigloyloxy-6,7-epoxytropane | C13H19NO3 | 238.3033 | 238.1438 | 0.1438 | 5 |  |
| 3-Hydroxy-6-tigloyloxytropane | C13H21NO3 | 240.3192 | 240.1594 | 0.1594 | 5 |  |
| 3-Tigloyloxy-6,7-dihydroxytropane | C13H21NO4 | 256.3186 | 256.1543 | 0.1543 | 5 |  |
| Cochlearine | C15H19NO3 | 262.3248 | 261.136 | 0.136 | 5 |  |
| Apoatropine | C17H21NO2 | 272.3627 | 272.1645 | 0.1645 | 5 |  |
| 3-Tigloyloxy-6-propionyloxy-tropane | C16H25NO4 | 296.3825 | 296.1856 | 0.1856 | 5 |  |
| Scopolamine | C17H21NO4 | 304.3615 | 304.1543 | 0.1543 | 5 |  |
| 3-Tropoyloxy-6-acetoxytropane | C19H25NO5 | 348.4142 | 348.1805 | 0.1805 | 5 |  |
| 3,7-Dihydroxy-6-(2′-methylbutyryl-oxy)-tropane | C13H23NO4 | 258.3345 | 258.1705 | 0.1705 | 5 |  |
| 3-Tigloyloxy-6-hydroxytropane | C13H21NO3 | 240.3192 | 240.1599 | 0.1599 | 5 |  |
| Withasomnine | C12H12N2 | 185.2455 | 185.2376 | 0.2376 | 5 |  |

****m/z measured for* [M+H]^+^ *adduct for all the compounds***

Annotation Levels:

Level 0 = Unambiguous 3D Structure: Isolated Compound including full stereochemistry, following natural products guidelines, determination of 3D structure

Level 1 = Confirmed structure by reference standard. MS, MS/MS, RT, reference standard

Level 2 = Probable structure (literature, library): Unambiguous matching literature or library MS/MS spectrum

Level 3 = Probable structure (experimental, in silico): Structure determined based on MS, MS/MS fragments sub-structure matching with experimental data or in silico fragmentation.

Level 4 = Tentative candidate: Class of structures based on MS, MS/MS, experimental data, but positional isomers cannot be distinguished

Level 5 = Unequivocal molecular formula based on MS, isotopes, adducts, RDBE

Level 6 = Exact mass of interest, no proposed structure or formula


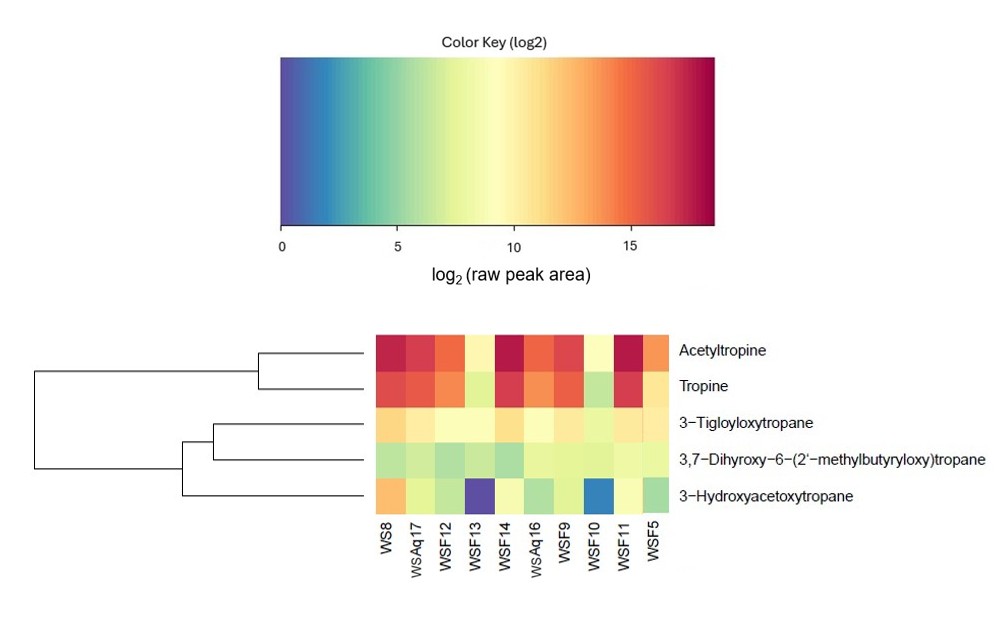


**Figure S8: Heat map comparison of selected tropane alkaloid peak areas detected using LC-HRMS/MS in the tested fractions.** Peaks were identified as shown shown in Table S5. Peak area data is presented as log_2_ raw peak area measured in counts per second.


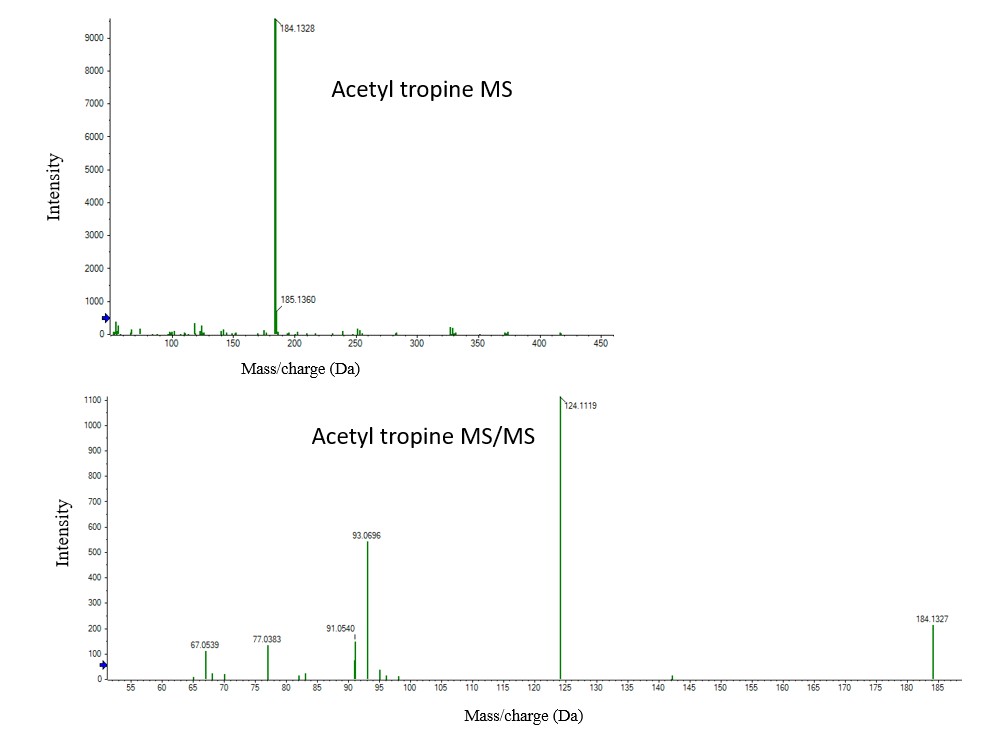


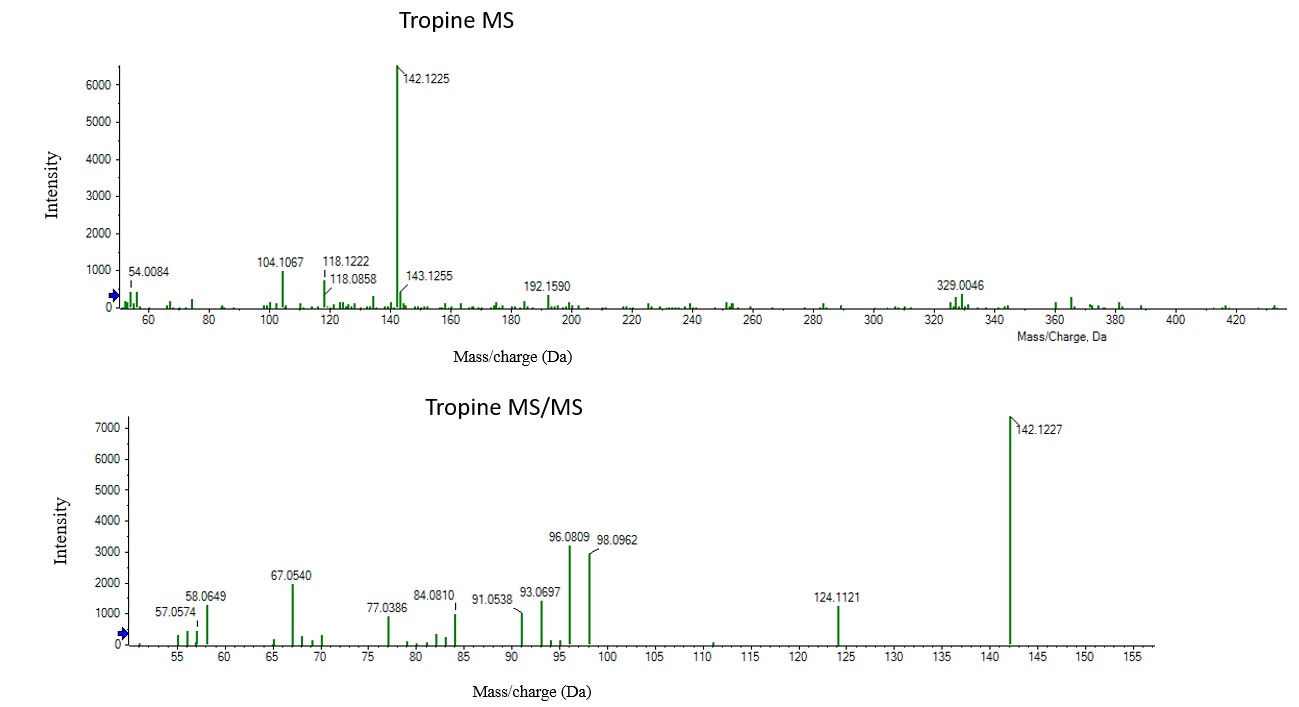


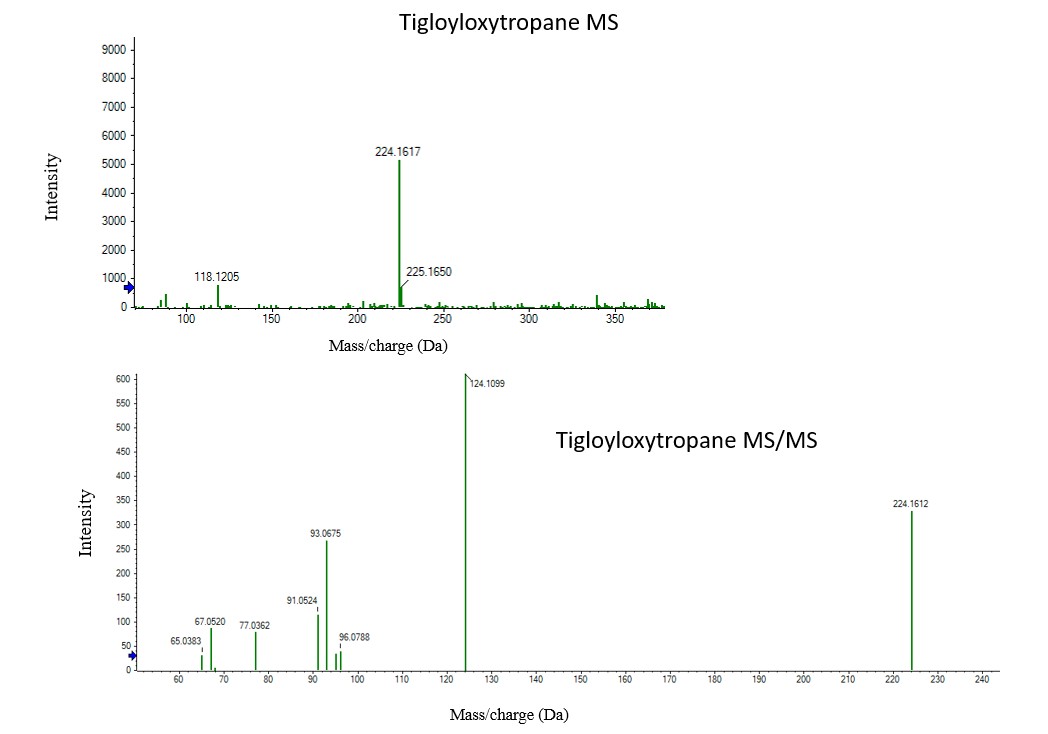


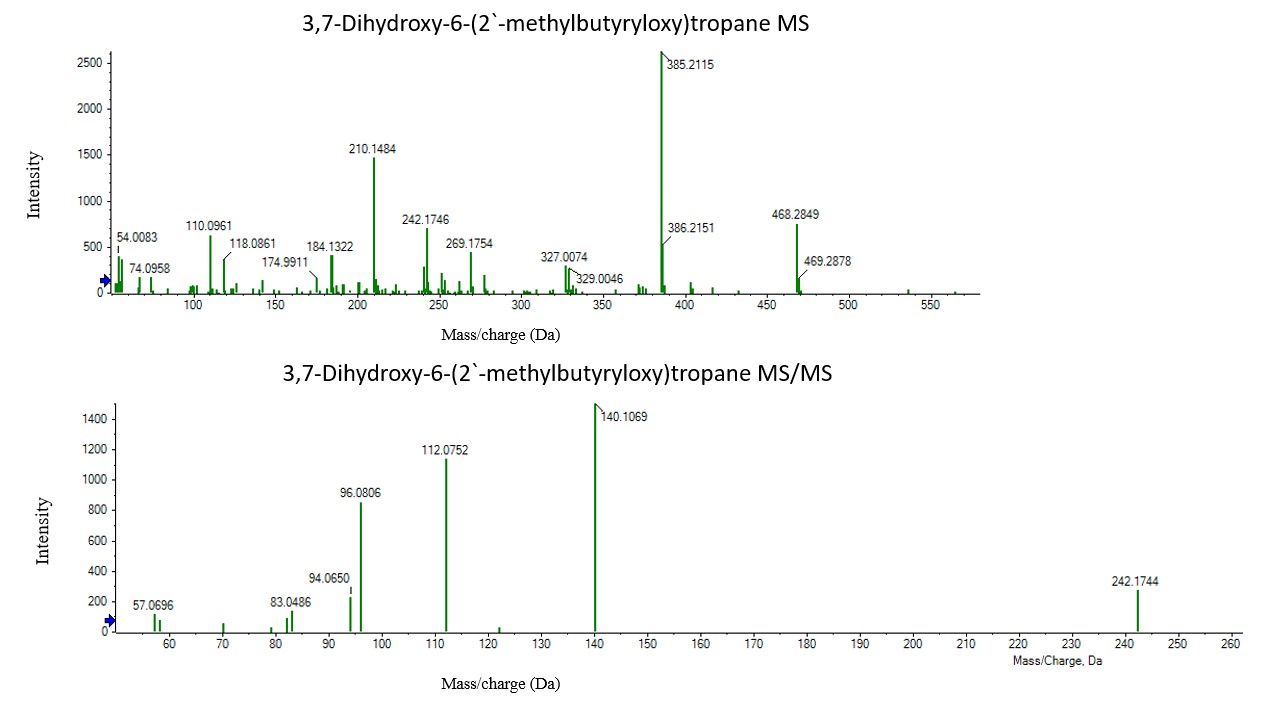


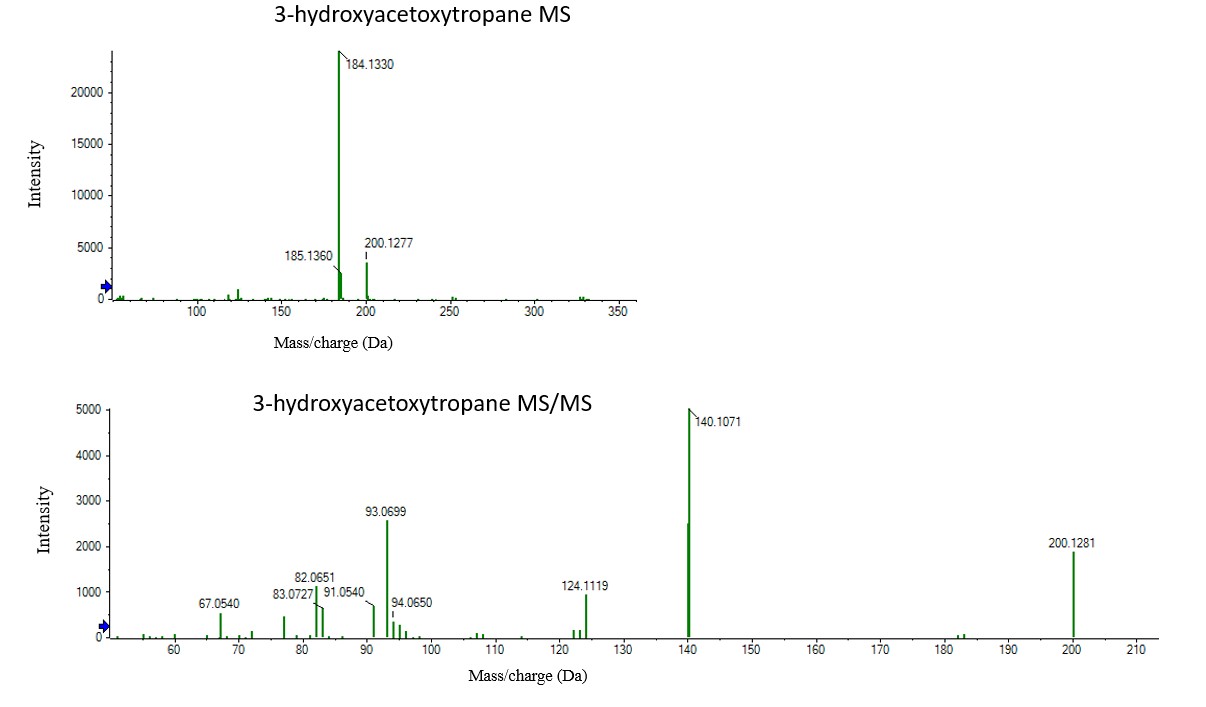


**Figure S9: MS1 And MS/MS Spectra of alkaloids shown in the heatmap (Figure S8)**

**Supplementary Section S1**

**Chemical Synthesis of Acetoxytropane isomers**

**S 1.1 Reagents**

For the acetoxytropane isomer synthesis, chemicals and solvents were purchased from ThermoFisher or Sigma Aldrich unless otherwise noted. Tropine (CAS 120-29-6) and its isomer exo-8-methyl-azabicylo[3.2.1]octan-3-ol (CAS 135-97-7) were purchased from Combi-Blocks.

**S 1.2 Chemical synthesis**

The chemical syntheses were performed by the OHSU Medicinal Chemistry Core. Acetyltropine and its stereoisomer acetyl exotropine were synthesized by acetylation of tropine and exo-8-methyl-azabicylo[3.2.1]octan-3-ol, respectively. Starting tropine isomer (50 mg, 0.35 mmol) and chloroform (1.5 mL) was added to a vial, which was sealed, flushed with argon, then cooled to 0^ο^C. Acetyl chloride (0.13 mL, 1.8 mmol) was added, the reaction was warmed to RT, then refluxed for 18 hr. After completion the reaction was concentrated and dried under vacuum to give the product, which was used without further purification. Liquid chromatography/mass spectrometry of the products was carried out on an Agilent 1260 Infinity II coupled to a ThermoFisher LTQ Velos.

**S1.3 Structural verification:**

Liquid chromatography/mass spectrometry was carried out on an Agilent 1260 Infinity II coupled to a ThermoFisher LTQ Velos. 1D-NMR was carried out on a Bruker 400 MHz Avance NEO NanoBay spectrometer. Two-dimensional correlation spectroscopy to differentiate the two isomers was performed on a Bruker 700 MHz spectrometer.

**S.1.4 Results**

**Acetyl tropine HCl.** 50 mg (65% yield). ^1^H NMR (chloroform-d) δ 12.55 (s, 1H); 5.14 (t, 1H) 3.75 (s, 2H); 3.10 (d, 2H); 2.75 (s, 3H); 2.40 (d, 2H); 2.24 (d, 2H); 2.08 (s, 3H); 2.00 (d, 2H).

**Acetyl *exo*-tropine HCl.** 65 mg (85% yield). ^1^H NMR (chloroform-d) δ 12.84 (s, 1H); 5.06 (m, 1H); 3.80 (s, 2H); 2.74 (m, 5H); 2.27 (m, 2H); 2.13 (m, 4H); 2.05 (s, 3H).

Both products were observed by mass spectrometry at the expected M/z of 184 (M+1).

NOESY 2D NMR data confirms the stereochemical identity of the two isobars. In acetyl exo-tropine, clear nuclear Overhauser enhancements (NOEs) are observed between H8 and the axial protons H4′ and H5′, indicating close spatial proximity consistent with the endo configuration of the tropine ring. These key NOE cross-peaks are absent in the acetyltropine spectrum, where the H8 configuration places out of range for dipolar coupling with H4′ and H5′. The differential NOESY correlations serve as definitive evidence for assigning the correct stereochemistry.

A)


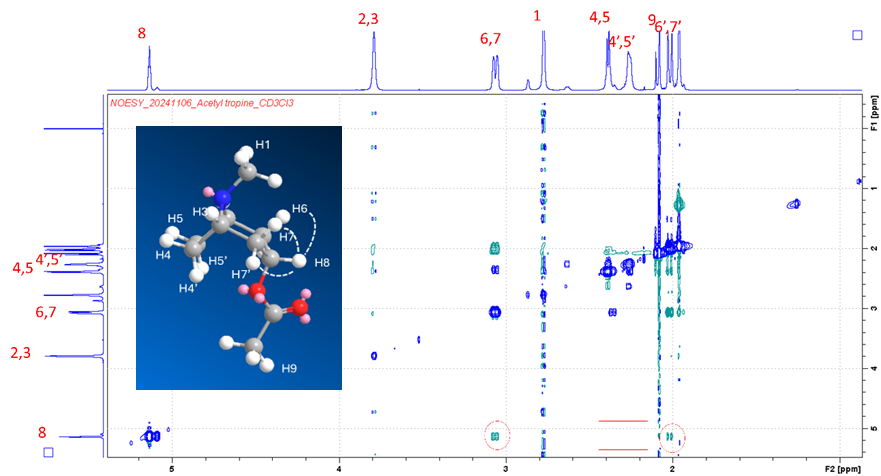


B)


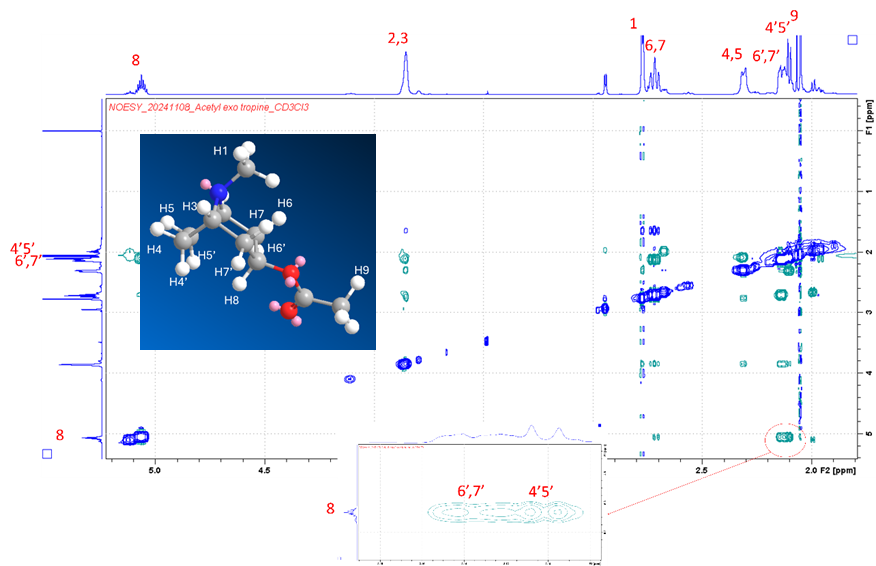


**Figure S10**: 2D NMR (NOESY) data confirming the stereochemistry of the two isomers A) acetyl tropine, B) acetyl exo-tropine. Correlation of H8 with H4’ and H5’ are key to determining identity of the β-isomer.

**Supplementary Section S2**

**Method development for accurate quantification of acetyltropine in extracts and fractions by LC-MRM-MS**

**S2.1 Column selection for chromatography:**

A previously published method for separation and accurate measurement of withanolide isobars was first applied to the analysis of extracts and fractions of WS. That method uses a phenyl-3 column and successfully separates four known isobaric withanolides. However, alkaloids are barely retained using Phenyl-3 as stationary phase and elute early using this method. A rever**s**ed phase (RP) C18-method was developed that showed sufficient retention for alkaloids. A binary gradient elution method was developed consisting of solvent A, water (containing 0.1 % FA) and acetonitrile (containing 0.1 % FA. (Lai, Wu et al, 2019).

We deviated from the published method by changing the total runtime to 17 minutes and the gradient to achieve better and repeatable peak separation. This method successfully separated the acetyltropine and acetyl exotropine.

The isomers could be distinguished by LC-MRM-MS (Figure S8) with retention times of 1.4 (acetyl exotropine) and 1.8 (acetyltropine) respectively. The isomer found in BEN-WS-8 root and the active water extract WSAq17, fraction WSF-14 and alkaloidal extract WSF-5 was acetyltropine


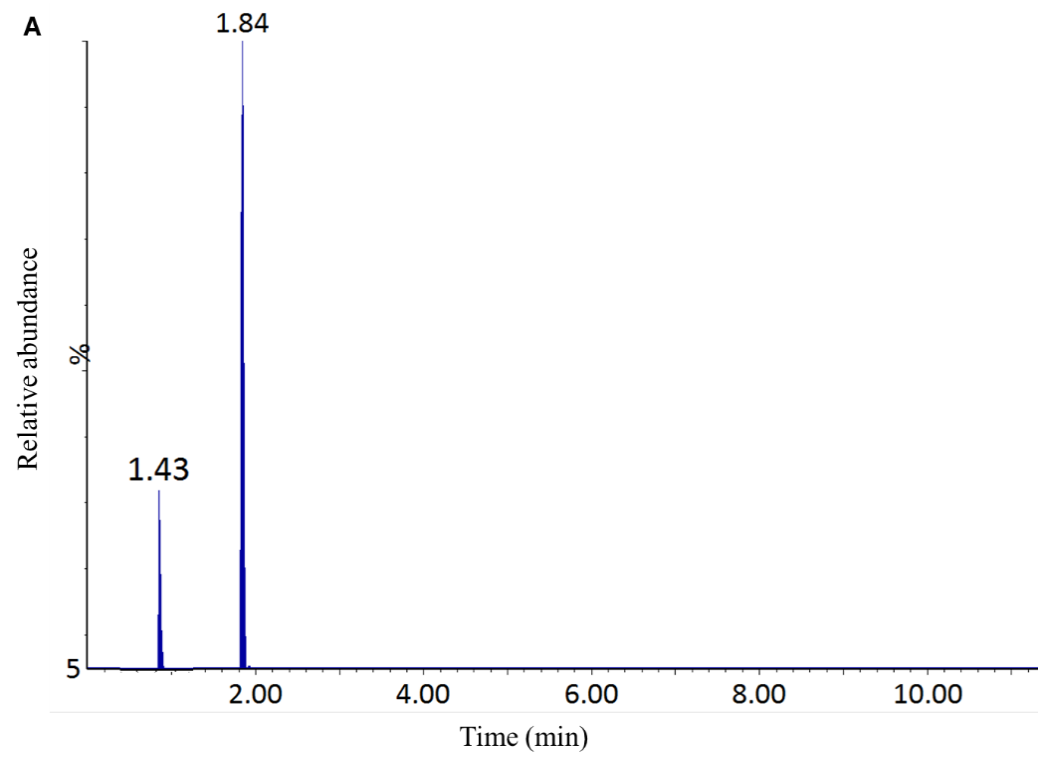


**Figure S11**: (A) Acetyltropine and acetyl exotropine separated by C18 LC-MRM-MS method as described.

**S2.2 Validation of LC-MRM-MS method: Linearity, linear dynamic range, and sensitivity**

The LC-MRM-MS method for acetyltropine quantification was validated in accordance with FDA Q2(R1) guidelines, assessing selectivity, linearity, precision, range, sensitivity (LOD, LOQ), and robustness. Calibration curves were generated using 16 standard concentrations spanning five orders of magnitude (0.0001–1 µg/mL), each injected in triplicate. A 1/x weighted linear regression model was applied using TargetLynx software. Method precision was evaluated through triplicate analyses of BEN–WS-8 by three independent analysts over five months. To monitor long-term analytical consistency, an extract of BEN-WS-8 root was employed as a quality control (QC) material. QC performance was visualized using Levey-Jennings plot (Figure S9), where deviations beyond ±2 SD triggered further inspection for systematic or random error. The method demonstrated good robustness for repeated measurements, and was selective for acetyltropine, with a limit of detection of 0.0001 µg/mL and limit of quantitation of 0.0003 µg/mL.


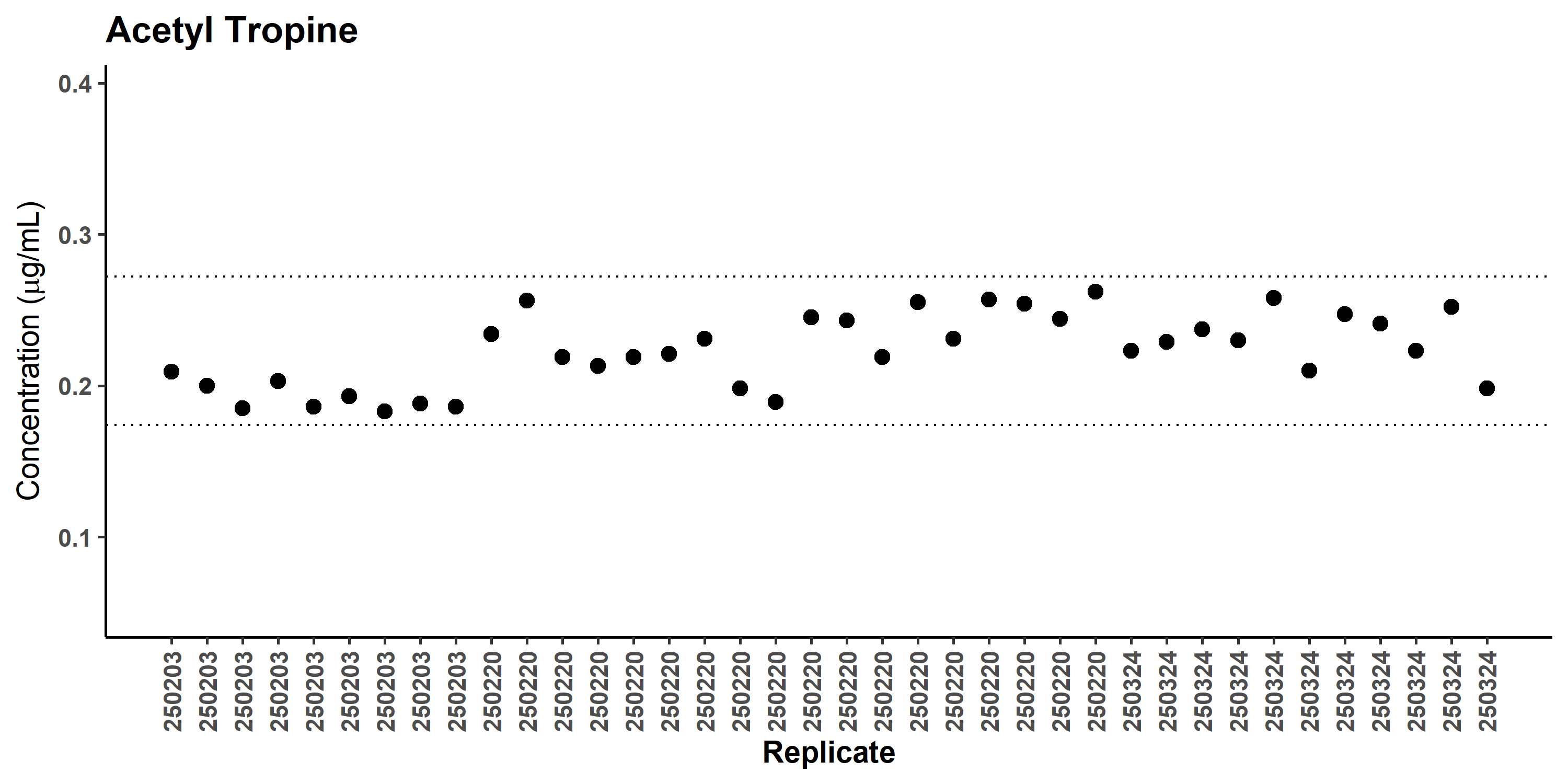


**Figure S12**: Levey-Jennings plots showing the reproducibility of this assay over three months. Two separate scientists performed the assay on multiple days. The dotted line represents ± 2 standard deviations.

**References:**

Guoyin Lai, Lijian Wu, Zhongda Lin, Liyi Lin, Dunming Xu, Zhigang Zhang and Meiling Lu (2019). High-Throughput Determination of Multiple Toxic Alkaloids in Food by UHPLC/MS/MS. Agilent Application note: Food Testing and Agriculture. July 31, 2019.

https://lcms.cz/labrulez-bucket-strapi-h3hsga3/application_alkaloids_high_throughput_lcmsms_5994_1125en_agilent_1bf39e132f/application-alkaloids-high-throughput-lcmsms-5994-1125en-agilent.pdf
