# Supplementary material for: Mouse neuronal dendritic complexity and resilience to stress-induced depression in Drosophila melanogaster are enhanced by Withania somnifera alkaloids
Source: J Ethnopharmacol. Author manuscript; Available in PMC 2026 Feb 5. (PMC12875680; doi:10.1016/j.jep.2025.120905)
Supplement: MMC1 [file NIHMS2126075-supplement-MMC1.docx]

**List of compounds studied**

Acetyl exotropine

Acetyltropine

Atropine d_5_

12-Deoxywithastramonolide

Tropine

Withaferin A

Withanolide A

Withanolide B

Withanone,

Withanoside IV

Withanoside V
